# Interactions among maternal smoking, breastfeeding, and offspring genetic factors on the risk of adult-onset hypertension

**DOI:** 10.1186/s12916-022-02648-y

**Published:** 2022-11-23

**Authors:** Jingjia Liang, Zuqiang Fu, Qian Liu, Yuehong Shen, Xin Zhang, Zhenkun Weng, Jin Xu, Wenxiang Li, Cheng Xu, Yong Zhou, Aihua Gu

**Affiliations:** 1 State Key Laboratory of Reproductive Medicine, Center for Global Health, School of Public Health, Nanjing Medical University, Nanjing, 211166 China; 2 Key Laboratory of Modern Toxicology of Ministry of Education, Center for Global Health, Nanjing Medical University, Nanjing, China; 3 School of Public Health, Southeast University, Nanjing, China; 4 CAS Key Laboratory of Tissue Microenvironment and Tumour, Shanghai Institute of Nutrition and Health, Shanghai Institutes for Biological Sciences, Chinese Academy of Sciences, Shanghai, 200031 China; 5 Key Laboratory of Stem Cell Biology, Institute of Health Sciences, Shanghai Institutes for Biological Sciences, Chinese Academy of Sciences & Shanghai Jiao Tong University School of Medicine, Shanghai, 200031 China

**Keywords:** Maternal smoking, Breastfeeding, Polygenic risk score, Adult-onset hypertension, Prospective cohort study

## Abstract

**Background:**

Previous studies have reported that maternal smoking during pregnancy and breastfeeding may affect the occurrence of hypertension, but whether early life factors modify the impact of the offspring’s genetic risk on hypertension is still unknown. The aim of this study was to investigate the relationships among maternal smoking and breastfeeding with adult-onset hypertension and the modified impact of offspring genetic susceptibility.

**Methods:**

This study included 437,185 participants from the UK Biobank who were initially free of hypertension and provided a prospective cohort of individuals aged 40 to 69 years. The association of maternal smoking during pregnancy and breastfeeding with hypertension was examined by using the Cox regression model. Then, a polygenic risk score (PRS) for hypertension was used to test the gene–environmental interaction on hypertension.

**Results:**

During a median follow-up period of 8.7 years, a total of 68,148 cases of hypertension were identified in this study. The hazard ratios (HRs) and 95% confidence intervals (CIs) of hypertension for maternal smoking and breastfeeding were 1.11 (1.09, 1.13) and 0.96 (0.94, 0.98), respectively. However, no evidence of an interaction between maternal smoking and breastfeeding was observed. Across all levels of genetic risk, including high genetic risk, maternal smoking and nonbreastfeeding had higher hypertension hazards than nonmaternal smoking and breastfeeding, respectively. The adjusted HRs (95% CIs) of hypertension were 1.80 (1.73, 1.87) in those who had high genetic predisposition plus maternal smoking and 1.67 (1.60–1.74) in those with nonbreastfeeding and high genetic risk. There were significant additive interactions between maternal smoking or breastfeeding and genetic factors on the incidence of hypertension.

**Conclusions:**

Maternal smoking and nonbreastfeeding were associated with a higher risk of hypertension in adulthood and may attenuate the risk of hypertension related to genetic factors. These results suggested that adherence to nonmaternal smoking and breastfeeding was associated with a lower risk of hypertension among participants with all gradients of genetic risk.

**Supplementary Information:**

The online version contains supplementary material available at 10.1186/s12916-022-02648-y.

## Background

Adverse intrauterine exposure may result in permanent developmental inhibition of the structure and function of the cardiovascular system and increase susceptibility to various cardiovascular metabolic diseases later in life [1, 2]. Recent studies have linked gestational exposure to maternal smoking to cardiovascular risk in offspring [3–8]. Maternal smoking during pregnancy is an established risk factor for the intrauterine environment and might influence postnatal blood pressure levels [7–9]. The observed link could be attributed to exposure to smoking-related substances, including toxins such as nicotine and carbon monoxide, which may induce vasoconstriction and affect foetal blood vessel development [10, 11]. However, most studies of cardiovascular outcomes in the offspring of smokers have been conducted on children and adolescents, and little is known about the long-term effects.

In addition, data on the early-life determinants of cardiovascular risk suggest that breastfeeding has a protective effect on cardiovascular health [12, 13]. Breastfeeding has been shown to reduce diastolic blood pressure and lipid levels in children [9, 14–18]. The beneficial effects of breastfeeding on blood pressure levels are suspected to be due to growth factors and hormones, inflammatory factors, oligosaccharides [19], and long-chain polyunsaturated fatty acids [20], which are not included in formula and may influence blood pressure. However, research on the association of maternal smoking and breastfeeding on blood pressure tends to focus only on their respective effects. The interaction of maternal smoking and breastfeeding on hypertension risk is still unknown.

Genome-wide association studies (GWAS) and mixed mapping studies in populations of European ancestry have identified more than 200 genetic loci [21, 22]. Polygenic risk scores have been used to obtain individual levels of overall genetic susceptibility to hypertension [23, 24]. A 1 standard deviation (SD) increase in systolic blood pressure (SBP) and diastolic blood pressure (DBP) polygenic risk scores (PRSs) resulted in 54% and 58% greater risks of early-onset hypertension, respectively [23]. In recent years, emerging evidence has revealed that genetic susceptibility might interact with early life factors on cardio-metabolic outcomes, including hypertension [25, 26]. However, whether exposure to maternal smoking or nonbreastfeeding may modify the impact of genetic predisposition on hypertension remains unknown.

Based on a cohort of ~500,000 persons from the UK Biobank, we extracted information on breastfeeding and maternal smoking during pregnancy and examined their interaction on offspring adult hypertension using Cox proportional risk models. Considering the importance of genetic factors [21], stratified analyses were carried out to explore the association of breastfeeding or maternal smoking during pregnancy with hypertension under different genetic risks.

## Methods

### Study design and population

The UK Biobank is a large, population-based, multicentre prospective cohort study that has collected a series of data on lifestyles, physical measures, biological samples, and health data [27, 28]. The original intention of the study was to provide resources to investigate the genetic and environmental determinants of complex chronic diseases in middle-aged and elderly people, which has been previously described in detail [27, 28]. In summary, ~0.5 million UK residents aged 40–69 years who registered with the UK National Health Service and lived < 25 miles from 1 of 22 research assessment centres across the UK were enrolled from 2006 to 2010. The baseline summary characteristics of the cohort can be viewed in the data showcase on UK Biobank’s website (www.ukbiobank.ac.uk). The UK Biobank study was approved by the research ethics committee of the UK Biobank, and all participants provided informed consent forms.

### Assessment of maternal smoking and breastfeeding

Information on “maternal smoking around birth (Field ID 1787)” and “breasted as a baby (Field ID 1677)” was collected based on the ACE touchscreen question. Participants were asked, “Did your mother smoke regularly around the time when you were born?” or “Were you breastfed when you were a baby?” The answer options were “yes”, “no” and “don’t know”. The relevant information can be found in the “Early life factors” (Field ID 100033) category of the UK Biobank’s website (https://biobank.ndph.ox.ac.uk/showcase/label.cgi?id=100033).

### Definition of the polygenic risk score

The detailed procedures for genotyping, imputation and quality control in the UK Biobank project have been described previously [29]. Based on the largest available hypertension genome-wide association study (GWAS) [21], we constructed a PRS with 797 single nucleotide polymorphisms (SNPs) (imputation quality score with INFO ≥ 0.3 and heterogeneity Cochran’s *Q* statistic19 filtered at *P* ≥ 1 × 10^−4^). All SNPs included in the current study were available in the UK Biobank imputed dataset. The related information is presented in Additional file 1: Table S1.

A weighted method was applied to calculate the hypertension PRS. According to the number of risk alleles, each SNP was redefined as 0, 1, or 2. The PRS values were calculated using the formula: PRS = *β*_1_ × SNP_1_ + *β*_2_×SNP_2_ + … +*β*_*k*_ × SNP_*k*_ + … + *β*_*n*_×SNP_*n*,_ where *n* is the total number of SNPs and *β*_*k*_ is the value of the natural logarithm of the odds ratio for hypertension associated with SNP_*k*_. The effect size estimates of *β*_*k*_ were obtained from a previous study [21]. The participants were divided into three levels of low (lowest tertile), intermediate (middle tertile), and high (highest tertile) genetic risk of hypertension based on the PRS distributions among noncases, which was shown to be effective [30, 31].

### Ascertainment of outcomes

Hypertension existing at baseline was defined using self-reported information and data from hospital episode statistics with the date of event preceding the date of attendance at the assessment centre. Hypertension was confirmed by hospital admissions with International Classification of Diseases, 9th and 10th Revision codes of 401. X, I10, 20002, 6150, and 6177 [32]. Follow-up times for individuals were calculated as the number of days from the assessment date until the hypertension incident or censorship date according to the origin of the hospital date. Diagnoses and dates for hospital admissions were determined through record linkage to the Health Episode Statistics in England, Scotland and Wales.

### Definition of covariates

Additional information was collected by self-reporting or historical diagnoses from national hospital registers. We included age; sex (male/female); UK Biobank assessment centre; maternal smoking around birth (yes/no); breastfed as a baby (yes/no); Townsend Deprivation Index (TDI); body mass index (BMI, normal/overweight/obesity); physical activity (metabolic equivalent task, MET-min/week); smoking status (never/previous/current); alcohol consumption (never/previous/current); diabetes at baseline (yes/no); and cardiovascular disease at baseline (yes/no).

Based on items obtained from the short International Physical Activity Questionnaire (IPAQ), the number of metabolic equivalent task (MET) minutes was adopted to assess physical activity. Smoking and alcohol consumption status were grouped as never, previous or current. In addition, BMIs were generated from height and weight data, which were measured by trained nurses during the baseline assessments. BMIs were classified as normal (< 25 kg/m^2^), overweight (25–29.9 kg/m^2^), or obese (≥ 30 kg/m^2^) according to the World Health Organization (WHO) criteria. Systolic blood pressure (SBP) and diastolic blood pressure (DBP) were measured by trained nurses at baseline using standard procedures. All of the above procedures were conducted by skilled professionals, and the mean values of two automated or manual measurements were used.

### Analytical cohort

**Fig. 1:**
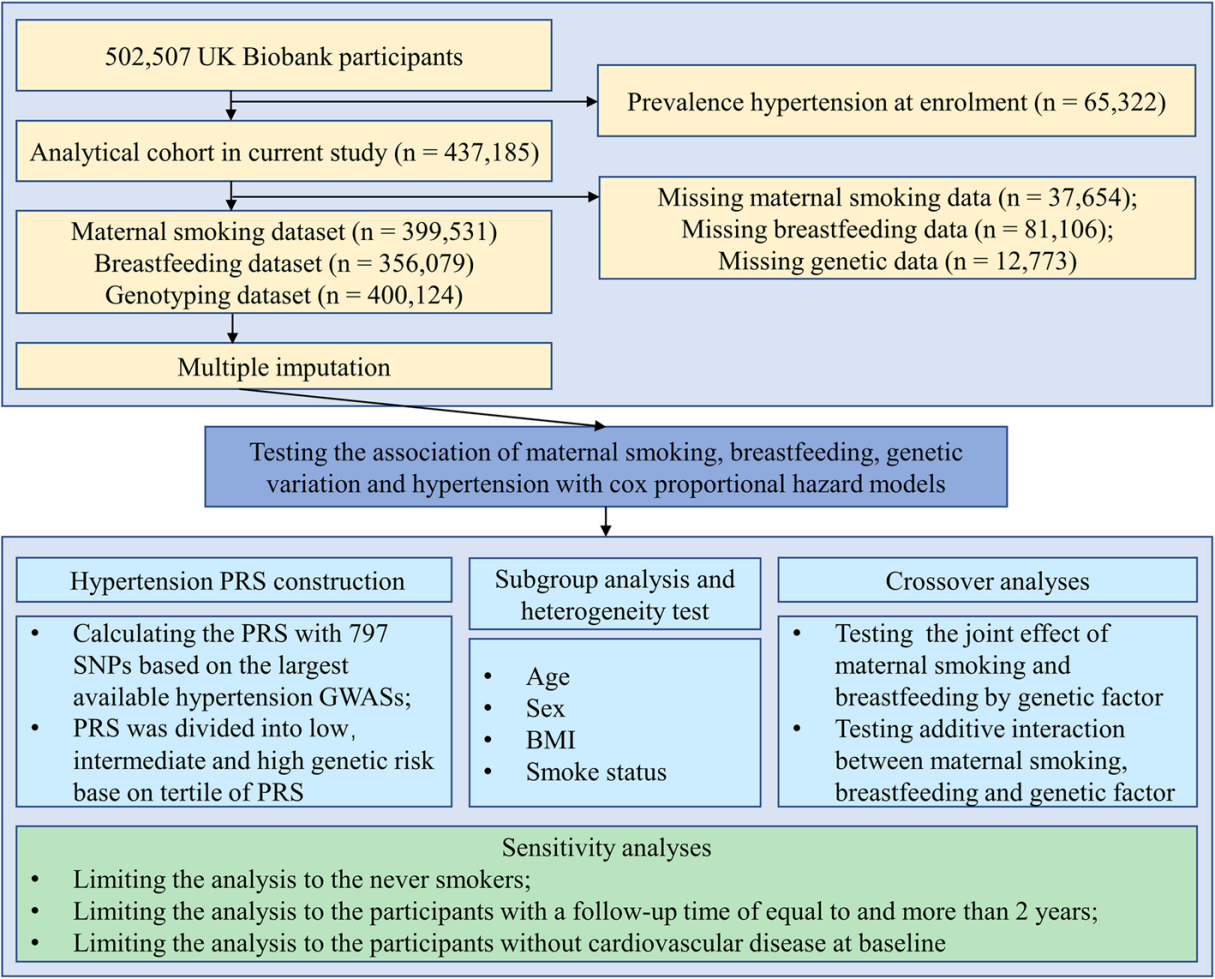
Flow diagram for inclusion of participants. CAD, coronary artery disease; PRS, polygenic risk score; GWAS, genome-wide association study; and SNPs, single nucleotide polymorphisms

Among the 502,507 individuals with available data for the current study, participants were excluded for the following reasons: (1) prevalent hypertension cases (defined by the date of a hypertension event preceding the date of enrolment or self-reported history of hypertension at baseline); (2) missing relevant exposure data such as maternal smoking or breastfeeding; or (3) missing genotyping data. Finally, there were 437,185 participants with complete hypertension follow-up data after excluding prevalent hypertension. Then, we included 399,531 and 356,079 participants to investigate the association between maternal smoking and breastfeeding with adult hypertension, respectively, after excluding participants with missing related exposure data. After excluding those who are missing all of exposure data, 318,425 participants involved in the analysis of the association of interaction between maternal smoking and breastfeeding with hypertension. Moreover, we further investigated the interaction of maternal smoking or breastfeeding and genetic factors (PRS) on hypertension when limiting participants to individuals with white British ancestry with genetic information available (*n* = 289,397, after further excluding participants who are missing genotyping data). A detailed flow diagram for the included participants is shown in Fig. 1. To rule out potential selection bias, we obtained descriptive statistics on the baseline information for those individuals with baseline hypertension or with missing covariates and did not find any obvious differences compared to the original participants (Additional file 1: Table S2).

### Statistical analysis

Descriptive statistics were obtained for exposures, outcomes, and covariates in the whole analytical cohort. The definition of survival time for each participant was the duration from the date of enrolment or self-reporting to the date of the hypertension event. Cox proportional hazard models were used to explore the associations between different exposures and adult-onset hypertension, and the results are shown as hazard ratios (HRs) and 95% confidence intervals (CIs). The proportional hazards assumption was examined using Schoenfeld residuals. The data generally met the conditions of the proportional hazards assumption for performing subsequent regression analyses (*P* > 0.05) (Additional file 1: Fig. S1).

To clearly illustrate the relationships among maternal smoking, breastfeeding, genetic variation and adult-onset hypertension, we constructed the following models: model 1: crudely adjusted for age and sex; model 2: model 1 further adjusted for race, TDI, alcohol consumption, smoking status, BMI, physical activity, and diabetes at baseline (yes/no); and model 3: model 1 further adjusted for TDI, alcohol consumption, smoking status, BMI, physical activity, and diabetes at baseline (yes/no). The main analysis was performed based on model 2, and we used model 3 instead when considering genetic factors. Due to possible confounding factors, we performed subgroup analyses by age (< 60 and ≥ 60 years, defined as elderly individuals by the WHO [33]), sex (male/female), BMI (normal/overweight/obesity), and smoking status (never/previous/current) using a Cox proportional hazards model adjusted for age, sex, race, TDI, alcohol consumption, smoking status, BMI, physical activity, and diabetes at baseline (the stratified factor in each stratum was excluded). Then, we conducted a heterogeneity analysis to assess whether the effect modifications between subgroups were statistically significant.

The interaction of specific exposures on hypertension was evaluated based on model 2 by (1) taking the maternal smoking status as a subgroup to assess the association of breastfeeding with hypertension; (2) taking the maternal smoking status as a subgroup to assess the association of own smoking status with hypertension; (3) taking the maternal smoking status as a subgroup to assess the difference in sex status on hypertension; and (4) taking participants who were not exposed to maternal smoking but were breastfed as a reference to assess the HR of increased risk factors on hypertension.

Sensitivity analyses were conducted to examine the robustness of the results based on model 3 by (1) limiting nonsmokers to avoid confounding effects caused by self-smoking; (2) limiting participants with follow-up times equal to or greater than 2 years to capture more hypertension events; and (3) limiting participants without prevalent CVD to avoid the possible confounding bias generated by CVD.

Additionally, we tested gene–environmental interactions by setting variable cross-product terms of the environmental exposures (maternal smoking and breastfeeding) with the hypertension PRS in model 3. The additive interaction term was constructed from two indices: the relative excess risk due to the interaction (RERI) and the attributable proportion (AP) [34]. The 95% CIs of the RERI and AP were determined by drawing 5000 bootstrap samples from the tested dataset [35], and when the CIs of the RERI and AP included 0, there was no interaction.

All missing covariates were included in the imputation equation. The missing category variables were imputed with multiple imputations based on latent class (MILC), and the missing continuous variables (e.g., TDI and physical activity) were imputed with multivariate imputation by chained equation (MICE) by using predictive mean matching [36, 37]. All analyses were performed using STATA software (Version 15.1) and R software (Version 4.1.1), and two-sided *P* values < 0.05 were considered statistically significant.

## Results

### Baseline characteristics of participants

The baseline characteristics of the final analytical cohort are presented in Table 1. The mean age at enrolment was 56.0 (SD, 8.1) years old; 44.4% were male; and 94.2% were individuals with white British ancestry. The mean TDI score, BMI, and MET at baseline were − 1.4, 27.2 kg/m^2^, and 2531.7 min/week, respectively. In brief, participants who suffered from hypertension were more likely to be obese and engaged in less physical activity. Additionally, participants with hypertension had a higher prevalence of CVD and diabetes. Hypertensive patients had higher maternal smoking rates and genetic risks but lower breastfeeding rates.

**Table 1:** Descriptive characteristics of participants in the UK Biobank study by hypertension

Characteristic	Incident hypertension		
	No (*n* = 369,037)	Yes (*n* = 68,148)	P*
Age (years, mean ± SD)	55.2 ± 8.1	60.1 ± 6.9	< 0.001
Sex, male (*n*, %)	158,626 (43.0)	35,676 (52.4)	< 0.001
Race, White (*n*, %)	348,077 (94.3)	63,621 (93.4)	< 0.001
TDI (mean ± SD)	− 1.4 ± 3.0	− 1.1 ± 3.2	< 0.001
BMI (kg/m^2^, mean ± SD)	26.8 ± 4.5	29.2 ± 5.1	< 0.001
BMI (kg/m^2^, *n*, %)			< 0.001
Normal (< 25 kg/m^2^)	138,021 (37.4)	13,258 (19.5)	
Overweight (25 to 29.9 kg/m^2^)	156,150 (42.3)	29,324 (43.0)	
Obesity (≥ 30 kg/m^2^)	73,212 (19.8)	25,103 (36.8)	
Missing value	1,654 (0.5)	463 (0.7)	
Physical activity (MET, Min/week, mean ± SD)	2680.2 ± 2706.5	2591.1 ± 2733.2	< 0.001
Smoke status (*n*, %)			< 0.001
Never	33,020 (48.4)	210,703 (57.1)	
Previous	27,366 (40.2)	119,044 (32.3)	
Current	7,418 (10.9)	38,123 (10.3)	
Missing value	344 (0.5)	1,167 (0.3)	
Alcohol drinker status (*n*, %)			< 0.001
Never	15,300 (4.1)	3,646 (5.3)	
Previous	11,646 (3.2)	2,921 (4.3)	
Current	341,733 (92.6)	61,464 (90.2)	
Missing value	358 (0.1)	117 (0.2)	
Diabetes baseline (*n*, %)	10,258 (2.8)	7,483 (11.0)	< 0.001
Maternal smoking around birth (*n*, %)	97,156 (28.8)	19,130 (30.8)	< 0.001
Breasted as a baby (*n*, %)	40,031 (76.3)	216,424 (71.3)	< 0.001

Data are presented as the mean ± standard deviation (SD), numbers and percentages

*Abbreviations*: *TDI* Townsend Deprivation index, *BMI* body mass index, *MET* Metabolic Equivalent Task

*P values were obtained by *t*-tests or chi-square tests

### Association of maternal smoking and breastfeeding with hypertension

A total of 68,148 cases of hypertension were identified during a median follow-up of 8.7 years, and the associations between maternal smoking or breastfeeding and incident hypertension are shown in Table 2. In the full model adjustment, participants with maternal smoking had a higher risk of hypertension (adjusted HR = 1.11, 95% CI, 1.09–1.13, *P* < 0.001). Participants who were breastfed presented a lower risk of hypertension (adjusted HR = 0.96, 95% CI, 0.94–0.98, *P* = 8.46 ×10^−4^). However, after stratification by maternal smoking, the association between breastfeeding and hypertension was not significant (adjusted HR = 0.97, 95% CI, 0.93–1.01, *P* = 0.124 and adjusted HR = 0.97, 95% CI, 0.94–1.00, *P* = 0.084, respectively) (Additional file 1: Table S3). Subgroup analyses showed that only age significantly modified the association of maternal smoking or breastfeeding with hypertension risk (*P* for interaction < 0.05, Additional file 1: Table S4). However, as presented in Additional file 1: Table S5, we observed that male participants with maternal smoking exposure had a higher risk of hypertension than female participants without maternal smoking (adjusted HR =1.42, 95% CI, 1.38–1.46, *P* < 2 × 10^−16^). It is well known that cigarette smoking in adulthood exerts a hypertensive effect. We found that participants exposed to both maternal and personal smoking were associated with a higher risk of hypertension (adjusted HR =1.43, 95% CI, 1.37–1.51, *P* < 2 × 10^−16^) than participants without maternal and personal smoking histories (Additional file 1: Table S6). After stratification by sex, both males and females with maternal smoking during pregnancy and their own smoking history showed a higher risk of hypertension (Additional file 1: Table S7, all *P* < 0.05).

**Table 2:** Adjusted hazard ratio and 95% confidence intervals of hypertension by different environmental exposures. Case/Control means participants who had hypertension /had no hypertension

Category of exposure	No. of cases/controls	Follow-up time (years, mean ± SD)	Model 1		Model 2	
			HR (95% CI)	*P*-value	HR (95% CI)	*P*-value
Maternal smoking						
No	43,010/240,235	8.5 ± 1.7	Ref		Ref	
Yes	19,130/97,156	8.5 ± 1.7	1.17 (1.14, 1.20)	< 0.001	1.11 (1.09, 1.13)	< 0.001
Breastfeeding						
No	12,420/87,204	8.3 ± 2.0	Ref		Ref	
Yes	40,031/216,424	8.1 ± 2.2	0.97 (0.94, 1.00)	0.088	0.96 (0.94, 0.98)	8.46E−04

Model 1, age (continuous), sex (male, female)

Model 2, adjusted for age (continuous), sex (male, female), race (White/Mixed/Asian or Asian British/Black or Black British), UK Biobank assessment centre, Townsend Deprivation index (continuous), alcohol consumption (never, previous, current, missing), smoking status (never, previous, current, missing), body mass index (< 25 kg/m^2^, 25 to 29.9 kg/m^2^, ≥ 30 kg/m^2^, missing), physical activity (continuous), and diabetes at baseline (yes/no)

*Abbreviations*: *SD* standard deviation, *HR* hazard ratio, and *CI* confidence interval

**Fig. 2:**
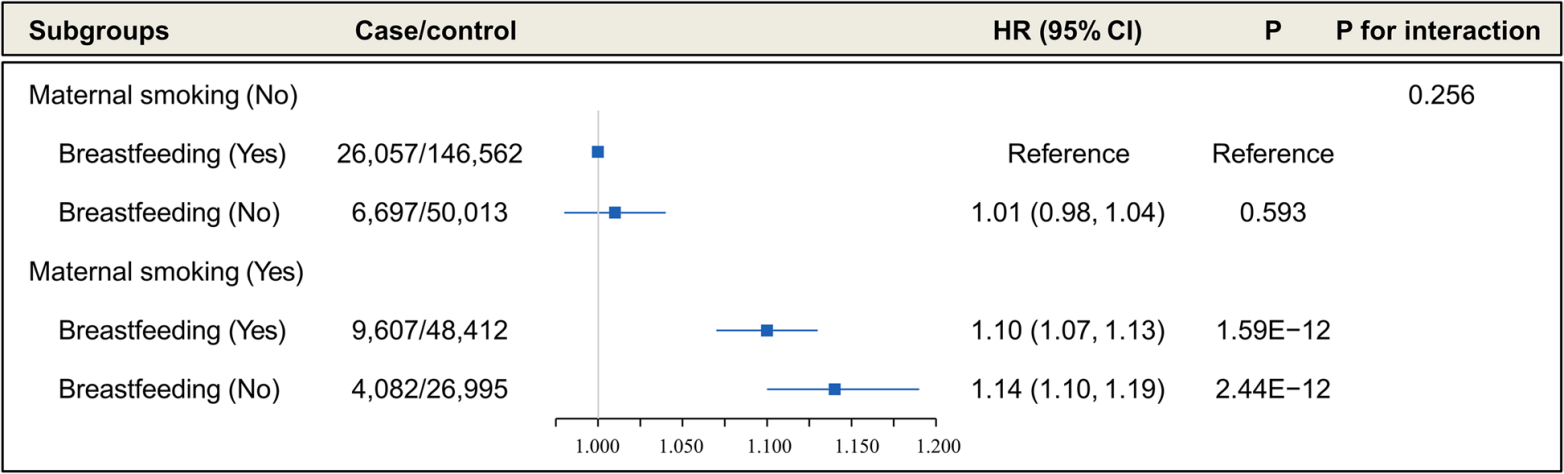
The joint association of maternal smoking and breastfeeding with the incidence of hypertension (participants without maternal smoking and no breastfeeding as a reference). Case/Control means participants who had hypertension /had no hypertension. Adjusted for age, sex, Townsend Deprivation Index (TDI), alcohol consumption, smoking status, BMI, physical activity, and diabetes at baseline

Figure 2 shows the joint association of maternal smoking and breastfeeding on hypertension. We found that participants with maternal smoking and no breastfeeding were associated with a higher risk of hypertension than those who were not exposed to maternal smoking but received breastfeeding (adjusted HR =1.14, 95% CI, 1.10–1.19, *P* = 2.44 × 10^−12^). However, no evidence of an interaction between maternal smoking and breastfeeding was observed (*P* for interaction = 0.256).

### Joint associations of maternal smoking, breastfeeding and genetic risk with hypertension

As reported in a previous study [23], in the analysis of genetic categories and hypertension risk, we confirmed that a higher PRS (genetic risk) was significantly associated with an increased risk of hypertension (all *P* < 2 × 10^−16^), and a per unit increase in PRS was significantly associated with a 7% increased risk of hypertension (adjusted HR = 1.07, 95% CI, 1.07–1.07, *P* < 2 × 10^−16^) (Additional file 1: Table S8).

Table 3 shows the joint associations of maternal smoking, breastfeeding and genetic risk with hypertension. For participants who had low genetic risk combined with nonmaternal smoking, the adjusted HRs (95% CIs) of hypertension were 1.40 (1.34, 1.46) and 1.80 (1.763, 1.87) in those who had intermediate and high genetic predisposition plus maternal smoking, respectively. In addition, compared to those with low genetic risk and breastfeeding, participants with low genetic risk and nonbreastfeeding were not associated with the risk of hypertension, but nonbreastfeeding was associated with higher hypertension risks compared with breastfeeding at intermediate genetic risk (adjusted HR = 1.37, 95% CI, 1.31–1.43, *P* < 0.001) as well as high genetic risk (adjusted HR = 1.67, 95% CI, 1.60–1.74, *P* < 0.001). The sensitivity analyses also showed that the relationships among maternal smoking, breastfeeding, and genetic factors and the incidence of hypertension were robust after excluding participants with cardiovascular disease at baseline (Additional file 1: Table S9). Moreover, when restricting participants with follow-up times equal to or longer than 2 years, the results did not change appreciably (Additional file 1: Table S10). In addition, the results remained robust when limiting the analysis to individuals who had never smoked (Additional file 1: Table S11).

**Table 3:** The joint association of maternal smoking and breastfeeding on incident hypertension in participants with different genetic risk

Hypertension PRS (tertiles)	Category of exposure	HR (95% CI)	*P*	*P* for interaction
	Maternal smoking			
Low genetic risk	No	Ref	Ref	0.148
	Yes	1.14 (1.09, 1.19)	3.95E−08	
Intermediate genetic risk	No	1.29 (1.25, 1.33)	< 0.001	
	Yes	1.40 (1.34, 1.46)	< 0.001	
High genetic risk	No	1.66 (1.60, 1.71)	< 0.001	
	Yes	1.80 (1.73, 1.87)	< 0.001	
	Breastfeeding			
Low genetic risk	Yes	Ref	Ref	0.378
	No	1.04 (0.99, 1.09)	0.165	
Intermediate genetic risk	Yes	1.25 (1.21, 1.30)	< 0.001	
	No	1.37 (1.31, 1.43)	< 0.001	
High genetic risk	Yes	1.64 (1.59, 1.69)	< 0.001	
	No	1.67 (1.60, 1.74)	< 0.001	

Adjusted for age (continuous), sex (male, female), UK Biobank assessment centre, Townsend Deprivation index (continuous), alcohol consumption (never, previous, current, missing), smoking status (never, previous, current, missing), body mass index (< 25 kg/m^2^, 25 to 29.9 kg/m^2^, ≥ 30 kg/m^2^, missing), physical activity (continuous), and diabetes at baseline (yes/no), genotyping batch, and the first 4 genetic principal components

*Abbreviation*s: *HR* hazard rations, *CI* confidence interval, *PRS* polygenic risk score

There was no evidence of multiplicative interactions between genetic and maternal smoking or breastfeeding for hypertension (Table 3). However, a positive additive interaction between genetic risks and maternal smoking or breastfeeding on the incidence of hypertension is shown in Table 4. Compared to participants with nonmaternal smoking and low genetic risk, participants with maternal smoking and high PRSs had a 16% (RERI = 0.16, 95% CI, 0.10–0.23) increased risk of hypertension; the interaction of genetic variation and breastfeeding accounted for 13% (RERI = 0.13, 95% CI, 0.06–0.20) of hypertension in participants with nonbreastfeeding and genetic variation.

**Table 4:** Additive joint interaction for included environmental exposure and genetic categories on hypertension

Category of exposure^b^		Hypertension PRS (tertiles)^b^			
		Intermediate^a^		High^a^	
		RERI (95% CI)	AP (95% CI)	RERI (95% CI)	AP (95% CI)
Maternal smoking	Yes	Ref	Ref	Ref	Ref
	No	0.34 (0.28, 0.41)	0.35 (0.29, 0.41)	0.21 (0.13, 0.29)	0.16 (0.11, 0.22)
Breastfeeding	Yes	Ref	Ref	Ref	Ref
	No	0.32 (0.27, 0.38)	0.32 (0.28, 0.37)	0.13 (0.06, 0.20)	0.09 (0.04, 0.14)

Adjusted for age (continuous), sex (male, female), UK Biobank assessment centre, Townsend Deprivation index (continuous), alcohol consumption (never, previous, current, missing), smoking status (never, previous, current, missing), body mass index (< 25 kg/m^2^, 25 to 29.9 kg/m^2^, ≥ 30 kg/m^2^, missing), physical activity (continuous), diabetes at baseline (yes/no), genotyping batch, and the first 4 genetic principal components

*Abbreviations*: *RERI* relative excess risk due to interaction, *AP* attributable proportion due to interaction, *CI* confidence interval, *PRS* polygenic risk score

^a^Defined by polygenic risk score: low (lowest tertiles), intermediate (second tertiles) and high (highest tertiles)

^b^To estimate RERI and AP, the maternal non-smoking category, breastfeeding category and the lowest genetic risk (low PRS) groups were the reference categories

## Discussion

In this large, population-based cohort study, we found that maternal smoking during pregnancy was positively associated with hypertension risk in adulthood, with a higher risk observed for those with maternal smoking, and that breastfeeding was associated with a lower risk of hypertension in adulthood. However, there was no evidence of an interaction between maternal smoking and breastfeeding for hypertension. In addition, the association between hypertension and maternal smoking or breastfeeding was modified by individuals’ unique genetic susceptibility to hypertension. Hypertension risk can be worsened by maternal smoking or nonbreastfeeding in participants with a moderate to high genetic risk for hypertension.

In line with our results, most studies have showed that higher blood pressure in offspring was associated with prenatal smoking [9, 38, 39]. A cohort of Norwegian women aged 17–47 [40] and another study of 33,086 participants in the Nurses’ Mothers’ Cohort [8] found that self-reported exposure to tobacco smoke in utero was associated with hypertension in adult women. However, the results do not generalise directly to men. In our study, males with maternal smoking showed a higher risk of hypertension than females with maternal smoking. In addition, active smoking (i.e., exposure to self-smoking in adulthood) significantly increased the effect of maternal smoking on offspring hypertension. This result is consistent with previous evidence for a potential synergistic effect of gestational and adult risk factors on cardiometabolic disease [41–43]. Foetal nicotine exposure leads to obesity and weight gain, changes in the composition and function of perivascular adipose tissue, and increased blood pressure [44, 45], which can be further exacerbated by smoke exposure in adulthood. Additionally, poor cardiovascular fitness may also result from prolonged hypoxia in the foetuses of women who smoked during pregnancy [46].

Nonbreastfeeding was also associated with an increased risk of hypertension in adulthood in our study. Consistent with our study, a previous mother-to-child study of 377 couples assessed short-term and long-term breastfeeding for offspring blood pressures at age 7 and found that long-term breastfeeding was associated with significantly lower systolic and diastolic blood pressures than short-term breastfeeding [15]. In addition, a meta-analysis comprising 17 observational studies reported that a small reduction in diastolic blood pressure in later life was associated with breastfeeding [47], whose results are certainly questionable due to the high heterogeneity. Notably, breastfeeding at ages older than 10 months was found to be a risk factor for hypertension in children (17,007 participants aged 6–12 years) [48]. However, their study was limited to obese offspring.

We found no evidence of an association between hypertension and the interaction of maternal smoking and breastfeeding. This may be related to additional exposure to tobacco compounds in breast milk. Smoking is an addictive behaviour that is difficult to stop immediately. Mothers who smoke during pregnancy or are exposed to second-hand tobacco smoke often smoke or are exposed to second-hand tobacco smoke after delivery [49]. Therefore, nicotine and other compounds that are present in smoking, breastfeeding mothers can be transmitted to the foetus through milk, forming an indirect exposure process to the foetus [50]. However, it is clear that the combination of smoking and not breastfeeding during pregnancy increased the hypertension risk. Additional research is warranted to further explore the extent to which individuals with maternal smoking would benefit from breastfeeding in comparison with those with nonmaternal smoking.

Other findings of this study were that the associations of maternal smoking or breastfeeding and hypertension could modify the impact of genetic risk. This finding suggests that individuals whose genetic predisposition to hypertension is low may have a higher risk of hypertension merely when their mothers smoke during pregnancy. In addition, individuals with low genetic risk may lose their inherent protection if maternal smoking is accompanied by no breastfeeding. Thus, avoiding exposure to maternal smoking and providing adequate breastfeeding after delivery may play an important role in the primary prevention of cardiovascular-related diseases such as hypertension in the population as a whole, especially in individuals at high genetic risk.

The main advantages of this study are the large sample size and prospectively collected hypertension event data. The association of combined exposure to breastfeeding and maternal smoking during pregnancy with the risk of hypertension was studied. In addition, this study stratified PRSs using a wide range of hypertension-related SNP information. The results of the analysis suggest that people who were exposed to maternal smoking during pregnancy, who were not breastfed or who have high PRSs need to be aware of the risk of hypertension. Factors such as breastfeeding and smoking during pregnancy may serve as predictive models for hypertension.

There are several limitations to this study. First, the UK Biobank participants were predominantly white, which limits the generalizability of the results to other ethnic groups. Second, the data for maternal smoking during pregnancy and breastfeeding were self-reported retrospectively, which can lead to recall bias. However, this might not be a significant problem because (1) previous studies have confirmed that reports by offspring of maternal smoking during pregnancy and breastfeeding are reasonably valid when compared to the mothers’ own reports [51, 52]; (2) in this study, the responses to maternal smoking status and breastfeeding were highly correlated (Cohen *κ* coefficient > 0.90) in subgroups of approximately 20,000 and 10,000 subjects who were assessed twice after the first and second follow-up; and (3) the proportion of maternal smoking in our study (29.1%) was close to the estimated prevalence of smoking during pregnancy in the UK (23.3%) [53]. Third, data on the extent or duration of maternal smoking during pregnancy, lactation, and second-hand smoke exposure are not available in the UK Biobank, which could be an area for future research. Fourth, genetic information for the original samples could not be obtained, and the overlapping populations of UKB could not be excluded from the calculations, which may result in a substantial inflation of the association between PRSs and disease outcomes when exploring the relationship between genetic variation and disease outcomes when exploring the relationship between genetic variations and disease. However, as in previous studies [54, 55], we aimed to investigate the modification of maternal smoking or breastfeeding on the impact of genetic susceptibility on hypertension risk. The hypertension PRSs in our study were identified as an instrumental variable and were used to reflect an individual's genetic risk. Finally, the lack of information on the frequency and intensity of maternal smoking and on the duration of exclusive breastfeeding and the availability of complementary foods for infants is also a limiting factor. More detailed studies of maternal smoking are needed in the future.

## Conclusions

In summary, based on a large cohort study, we found that participants who were exposed to maternal smoking during pregnancy or who were not breastfeeding had higher risks of high blood pressure in adulthood. A higher genetic risk for hypertension is also associated with a higher risk of developing hypertension. Notably, individuals with low genetic risk still need to pay attention to the risk of hypertension if they were exposed to maternal smoking during pregnancy or nonbreastfeeding. Additional efforts should be made to clarify the role of maternal smoking and breastfeeding in the aetiology of adult-onset hypertension.

## Supplementary Information

**Additional file 1: Table S1.** The main information for genetic variants associated with hypertension in the UK biobank. **Table S2.** Descriptive characteristics of participants in the UK Biobank study by hypertension. **Table S3.** The association of breastfeeding and maternal smoking on hypertension. **Table S4.** Subgroup analysis for the association of hypertension and maternal smoking or breastfeeding by specific characteristics. **Table S5.** The association of maternal smoking and sex with hypertension by sex (*n* = 399,531). **Table S6.** The association of maternal smoking and own smoking history with hypertension (*n* = 318,425). **Table S7.** The association of maternal smoking and own smoking history with hypertension by sex (*n* = 398,181). **Table S8.** Adjusted hazard ratios and 95% confidence intervals for hypertension polygenic risk scores with the risk of hypertension (*n* = 400,124). **Table S9.** The association of maternal smoking and breastfeeding with hypertension in participants with different genetic risks after excluding participants with cardiovascular disease at baseline (*n* = 283,057). **Table S10.** The association of maternal smoking and breastfeeding on hypertension in participants with different genetic risks after excluding participants with follow-up times less than 2 years in the UK Biobank (*n* = 278,873). **Table S11.** The association of maternal smoking and breastfeeding with hypertension in participants with different genetic risks among participants who never smoked (*n* = 162,439). **Figure S1.** The proportional hazards assumption using Schoenfeld residuals.

## Data Availability

The data that support the findings of this study are available from UK Biobank project site, subject to registration and application process. This research has been conducted using the UK Biobank resource under application number 55858. Further details can be found at https://www.ukbiobank.ac.uk.

## Abbreviations

AP: The attributable proportion
BMI: Body mass index
CIs: 95% confidence intervals
DBP: Diastolic blood pressure
GWAS: Genome-wide association studies
HRs: Hazards ratios
IPAQ: International Physical Activity Questionnaire
MET: Metabolic equivalent task
MICE: Multivariate imputation by chained equation
MILC: Multiple imputation based on latent class
PRS: Polygenic risk score
RERI: The relative excess risk due to the interaction
SBP: Systolic blood pressure
SNPs: Single nucleotide polymorphisms
TDI: Townsend Deprivation Index
WHO: World Health Organization

## References

[1] Taal HR, de Jonge LL, van Osch-Gevers L, Steegers EA, Hofman A, Helbing WA, van der Heijden AJ, Jaddoe VW (2013). Parental smoking during pregnancy and cardiovascular structures and function in childhood: the Generation R Study. Int J Epidemiol.

[2] Burton GJ, Fowden AL, Thornburg KL (2016). Placental origins of chronic disease. Physiol Rev.

[3] Yu Y, Arah OA, Liew Z, Cnattingius S, Olsen J, Sorensen HT, Qin G, Li J (2019). Maternal diabetes during pregnancy and early onset of cardiovascular disease in offspring: population based cohort study with 40 years of follow-up. BMJ.

[4] Power C, Atherton K, Thomas C (2010). Maternal smoking in pregnancy, adult adiposity and other risk factors for cardiovascular disease. Atherosclerosis.

[5] Mamun AA, O'Callaghan MJ, Williams GM, Najman JM (2012). Maternal smoking during pregnancy predicts adult offspring cardiovascular risk factors - evidence from a community-based large birth cohort study. PLoS One.

[6] Leybovitz-Haleluya N, Wainstock T, Landau D, Sheiner E (2018). Maternal smoking during pregnancy and the risk of pediatric cardiovascular diseases of the offspring: A population-based cohort study with up to 18-years of follow up. Reprod Toxicol.

[7] Hogberg L, Cnattingius S, Lundholm C, D'Onofrio BM, Langstrom N, Iliadou AN (2012). Effects of maternal smoking during pregnancy on offspring blood pressure in late adolescence. J Hypertens.

[8] de Jonge LL, Harris HR, Rich-Edwards JW, Willett WC, Forman MR, Jaddoe VW, Michels KB (2013). Parental smoking in pregnancy and the risks of adult-onset hypertension. Hypertension (Dallas, Tex : 1979).

[9] Lawlor DA, Najman JM, Sterne J, Williams GM, Ebrahim S, Davey Smith G (2004). Associations of parental, birth, and early life characteristics with systolic blood pressure at 5 years of age: findings from the Mater-University study of pregnancy and its outcomes. Circulation.

[10] Tuthill DP, Stewart JH, Coles EC, Andrews J, Cartlidge PH (1999). Maternal cigarette smoking and pregnancy outcome. Paediatr Perinat Epidemiol.

[11] Taylor L, Kelly B, Leeson P (2010). Maternal smoking and infant cardiovascular physiology: a mechanism of early cardiovascular disease development?. Hypertension.

[12] Martin RM, Ben-Shlomo Y, Gunnell D, Elwood P, Yarnell JW, Davey Smith G (2005). Breast feeding and cardiovascular disease risk factors, incidence, and mortality: the Caerphilly study. J Epidemiol Community Health.

[13] Gartner LM, Morton J, Lawrence RA, Naylor AJ, O'Hare D, Schanler RJ, Eidelman AI (2005). American Academy of Pediatrics Section on B: Breastfeeding and the use of human milk. Pediatrics.

[14] Plagemann A, Harder T (2005). Breast feeding and the risk of obesity and related metabolic diseases in the child. Metab Syndr Relat Disord.

[15] Hosaka M, Asayama K, Staessen JA, Ohkubo T, Hayashi K, Tatsuta N, Kurokawa N, Satoh M, Hashimoto T, Hirose T (2013). Breastfeeding leads to lower blood pressure in 7-year-old Japanese children: Tohoku Study of Child Development. Hypertens Res.

[16] Horta BL (2015). Loret de Mola C, Victora CG: Long-term consequences of breastfeeding on cholesterol, obesity, systolic blood pressure and type 2 diabetes: a systematic review and meta-analysis. Acta Paediatr.

[17] Fall CH, Borja JB, Osmond C, Richter L, Bhargava SK, Martorell R, Stein AD, Barros FC, Victora CG (2011). group C: Infant-feeding patterns and cardiovascular risk factors in young adulthood: data from five cohorts in low- and middle-income countries. Int J Epidemiol.

[18] Amorim Rde J, Coelho AF, de Lira PI, Lima Mde C (2014). Is breastfeeding protective for blood pressure in schoolchildren? A cohort study in northeast Brazil. Breastfeed Med.

[19] El-Khuffash A, Jain A, Lewandowski AJ, Levy PT (2020). Preventing disease in the 21st century: early breast milk exposure and later cardiovascular health in premature infants. Pediatr Res.

[20] Forsyth JS, Willatts P, Agostoni C, Bissenden J, Casaer P, Boehm G (2003). Long chain polyunsaturated fatty acid supplementation in infant formula and blood pressure in later childhood: follow up of a randomised controlled trial. BMJ.

[21] Evangelou E, Warren HR, Mosen-Ansorena D, Mifsud B, Pazoki R, Gao H, Ntritsos G, Dimou N, Cabrera CP, Karaman I (2018). Genetic analysis of over 1 million people identifies 535 new loci associated with blood pressure traits. Nat Genet.

[22] Simino J, Shi G, Weder A, Boerwinkle E, Hunt SC, Rao DC (2014). Body mass index modulates blood pressure heritability: the Family Blood Pressure Program. Am J Hypertens.

[23] Vaura F, Kauko A, Suvila K, Havulinna AS, Mars N, Salomaa V (2021). FinnGen, Cheng S, Niiranen T: Polygenic risk scores predict hypertension onset and cardiovascular risk. Hypertension.

[24] Luft FC (2021). Can single nucleotide polymorphisms beat Schnuffel?: Assessing hypertension polygenic risk scores. Hypertension.

[25] Curhan GC, Chertow GM, Willett WC, Spiegelman D, Colditz GA, Manson JE, Speizer FE, Stampfer MJ (1996). Birth weight and adult hypertension and obesity in women. Circulation.

[26] Song Q, Sun D, Zhou T, Li X, Ma H, Liang Z, Wang H, Cardoso MA, Heianza Y, Qi L (2021). Perinatal exposure to maternal smoking and adulthood smoking behaviors in predicting cardiovascular diseases: a prospective cohort study. Atherosclerosis.

[27] Collins R (2012). What makes UK Biobank special?. Lancet (London, England).

[28] Sudlow C, Gallacher J, Allen N, Beral V, Burton P, Danesh J, Downey P, Elliott P, Green J, Landray M (2015). UK biobank: an open access resource for identifying the causes of a wide range of complex diseases of middle and old age. PLoS Med.

[29] Bycroft C, Freeman C, Petkova D, Band G, Elliott LT, Sharp K, et al. Genome-wide genetic data on ~500,000 UK Biobank participants. bioRxiv. 2017. 10.1101/166298.

[30] Arthur RS, Wang T, Xue X, Kamensky V, Rohan TE (2020). Genetic factors, adherence to healthy lifestyle behavior, and risk of invasive breast cancer among women in the UK Biobank. J Natl Cancer Inst.

[31] Wang M, Zhou T, Song Y, Li X, Ma H, Hu Y, Heianza Y, Qi L (2021). Joint exposure to various ambient air pollutants and incident heart failure: a prospective analysis in UK Biobank. Eur Heart J.

[32] Larsson SC, Bäck M, Rees JMB, Mason AM, Burgess S (2020). Body mass index and body composition in relation to 14 cardiovascular conditions in UK Biobank: a Mendelian randomization study. Eur Heart J.

[33] WHO (2018). Ageing and health.

[34] Li R, Chambless L (2007). Test for additive interaction in proportional hazards models. Ann Epidemiol.

[35] Assmann SF, Hosmer DW, Lemeshow S, Mundt KA (1996). Confidence intervals for measures of interaction. Epidemiology.

[36] Azur MJ, Stuart EA, Frangakis C, Leaf PJ (2011). Multiple imputation by chained equations: what is it and how does it work?. Int J Methods Psychiatr Res.

[37] van Buuren S, Groothuis-Oudshoorn K (2011). mice: multivariate imputation by chained equations in R. J Stat Softw.

[38] Oken E, Huh SY, Taveras EM, Rich-Edwards JW, Gillman MW (2005). Associations of maternal prenatal smoking with child adiposity and blood pressure. Obes Res.

[39] Brion MJ, Leary SD, Lawlor DA, Smith GD, Ness AR (2008). Modifiable maternal exposures and offspring blood pressure: a review of epidemiological studies of maternal age, diet, and smoking. Pediatr Res.

[40] Cupul-Uicab LA, Skjaerven R, Haug K, Melve KK, Engel SM, Longnecker MP (2012). In utero exposure to maternal tobacco smoke and subsequent obesity, hypertension, and gestational diabetes among women in the MoBa cohort. Environ Health Perspect.

[41] Li Y, Ley SH, Tobias DK, Chiuve SE, VanderWeele TJ, Rich-Edwards JW, Curhan GC, Willett WC, Manson JE, Hu FB (2015). Birth weight and later life adherence to unhealthy lifestyles in predicting type 2 diabetes: prospective cohort study. BMJ.

[42] Li Y, He Y, Qi L, Jaddoe VW, Feskens EJ, Yang X, Ma G, Hu FB (2010). Exposure to the Chinese famine in early life and the risk of hyperglycemia and type 2 diabetes in adulthood. Diabetes.

[43] Eriksson JG, Yliharsila H, Forsen T, Osmond C, Barker DJ (2004). Exercise protects against glucose intolerance in individuals with a small body size at birth. Prev Med.

[44] Eskenazi B, Bergmann JJ (1995). Passive and active maternal smoking during pregnancy, as measured by serum cotinine, and postnatal smoke exposure. I. Effects on physical growth at age 5 years. Am J Epidemiol.

[45] Bruin JE, Gerstein HC, Holloway AC (2010). Long-term consequences of fetal and neonatal nicotine exposure: a critical review. Toxicol Sci.

[46] Zhang L (2005). Prenatal hypoxia and cardiac programming. J Soc Gynecol Investig.

[47] Martin RM, Gunnell D, Smith GD (2005). Breastfeeding in infancy and blood pressure in later life: systematic review and meta-analysis. Am J Epidemiol.

[48] Liang X, Xiao L, Luo Y, Xu J (2020). Prevalence and risk factors of childhood hypertension from birth through childhood: a retrospective cohort study. J Hum Hypertens.

[49] Havard A, Chandran JJ, Oei JL (2022). Tobacco use during pregnancy. Addiction.

[50] Ilett KF, Hale TW, Page-Sharp M, Kristensen JH, Kohan R, Hackett LP (2003). Use of nicotine patches in breast-feeding mothers: transfer of nicotine and cotinine into human milk. Clin Pharmacol Ther.

[51] Troy LM, Michels KB, Hunter DJ, Spiegelman D, Manson JE, Colditz GA, Stampfer MJ, Willett WC (1996). Self-reported birthweight and history of having been breastfed among younger women: an assessment of validity. Int J Epidemiol.

[52] Simard JF, Rosner BA, Michels KB (2008). Exposure to cigarette smoke in utero: comparison of reports from mother and daughter. Epidemiology.

[53] Lange S, Probst C, Rehm J, Popova S (2018). National, regional, and global prevalence of smoking during pregnancy in the general population: a systematic review and meta-analysis. Lancet Glob Health.

[54] Pazoki R, Dehghan A, Evangelou E, Warren H, Gao H, Caulfield M, Elliott P, Tzoulaki I (2018). Genetic predisposition to high blood pressure and lifestyle factors: associations with midlife blood pressure levels and cardiovascular events. Circulation.

[55] Fan M, Sun D, Zhou T, Heianza Y, Lv J, Li L, Qi L (2020). Sleep patterns, genetic susceptibility, and incident cardiovascular disease: a prospective study of 385 292 UK biobank participants. Eur Heart J.

