# Supplementary material for: Interactions among maternal smoking, breastfeeding, and offspring genetic factors on the risk of adult-onset hypertension
Source: BMC Med. 2022 Nov 23;20:454. doi: 10.1186/s12916-022-02648-y (PMC9694874; doi:10.1186/s12916-022-02648-y)
Supplement: Supplementary file 1 — Additional file 1: Table S1. The main information for genetic variants associated with hypertension in the UK biobank. Table S2. Descriptive characteristics of participants in the UK Biobank study by hypertension. Table S3. The association of breastfeeding and maternal smoking on hypertension. Table S4. Subgroup analysis for the association of hypertension and maternal smoking or breastfeeding by specific characteristics. Table S5. The association of maternal smoking and sex with hypertension by sex (n = 399,531). Table S6. The association of maternal smoking and own smoking history with hypertension (n = 318,425). Table S7. The association of maternal smoking and own smoking history with hypertension by sex (n = 398,181). Table S8. Adjusted hazard ratios and 95% confidence intervals for hypertension polygenic risk scores with the risk of hypertension (n = 400,124). Table S9. The association of maternal smoking and breastfeeding with hypertension in participants with different genetic risks after excluding participants with cardiovascular disease at baseline (n = 283,057). Table S10. The association of maternal smoking and breastfeeding on hypertension in participants with different genetic risks after excluding participants with follow-up times less than 2 years in the UK Biobank (n = 278,873). Table S11. The association of maternal smoking and breastfeeding with hypertension in participants with different genetic risks among participants who never smoked (n = 162,439). Figure S1. The proportional hazards assumption using Schoenfeld residuals. [file 12916_2022_2648_MOESM1_ESM.docx]

**Table S1.** The main information for genetic variants associated with hypertension in the UK biobank.

| Rs ID | Chr:position | Risk allele | Effect (beta) | Standard error | INFO | MAF | P value |
| --- | --- | --- | --- | --- | --- | --- | --- |
| rs880315 | 1:10796866 | T | -0.5076 | 0.0323 | 0.99 | 0.343 | 9.59E-56 |
| rs17030613 | 1:113190807 | A | -0.2847 | 0.0213 | 0.99 | 0.210 | 8.22E-41 |
| rs17367504 | 1:11862778 | A | 0.8968 | 0.0413 | 0.98 | 0.159 | 1.58E-104 |
| rs11585169 | 1:150572037 | A | 0.1557 | 0.0209 | 1.00 | 0.419 | 8.30E-14 |
| rs13796 | 1:154245917 | T | -0.1626 | 0.0257 | 1.00 | 0.135 | 2.44E-10 |
| rs3820068 | 1:15798197 | A | 0.2935 | 0.0387 | 0.91 | 0.196 | 3.31E-14 |
| rs3738633 | 1:16359827 | A | -0.1224 | 0.0177 | 0.99 | 0.450 | 4.96E-12 |
| rs2171690 | 1:164740099 | T | 0.1181 | 0.0174 | 0.98 | 0.465 | 1.19E-11 |
| rs7524019 | 1:167367193 | T | 0.1036 | 0.0174 | 1.00 | 0.495 | 2.60E-09 |
| rs7796 | 1:1684169 | C | 0.3385 | 0.0314 | 0.98 | 0.489 | 5.00E-27 |
| rs4656180 | 1:169113881 | A | -0.196 | 0.0215 | 0.94 | 0.347 | 6.63E-20 |
| rs12405515 | 1:172357441 | T | -0.1698 | 0.0174 | 1.00 | 0.430 | 1.92E-22 |
| rs12118102 | 1:176634724 | A | 0.3559 | 0.0459 | 1.00 | 0.052 | 8.45E-15 |
| rs150816167 | 1:179571862 | T | -0.2873 | 0.0446 | 0.96 | 0.043 | 1.17E-10 |
| rs41475048 | 1:183058452 | A | -0.1233 | 0.0202 | 0.99 | 0.254 | 9.93E-10 |
| rs882624 | 1:201735913 | T | -0.1571 | 0.0185 | 1.00 | 0.334 | 2.33E-17 |
| rs2169137 | 1:204497913 | C | 0.1588 | 0.0194 | 0.99 | 0.213 | 3.17E-16 |
| rs2629665 | 1:207220800 | A | -0.1193 | 0.0177 | 1.00 | 0.409 | 1.52E-11 |
| rs2761436 | 1:207919748 | T | 0.1767 | 0.0205 | 0.98 | 0.463 | 6.36E-18 |
| rs12408022 | 1:217718789 | T | 0.1483 | 0.0199 | 0.99 | 0.259 | 9.60E-14 |
| rs9431431 | 1:221358796 | A | -0.134 | 0.019 | 0.99 | 0.294 | 1.71E-12 |
| rs2760061 | 1:228191075 | A | 0.1771 | 0.0176 | 1.00 | 0.480 | 1.04E-23 |
| rs2004776 | 1:230848702 | T | 0.2513 | 0.0203 | 0.96 | 0.240 | 2.55E-35 |
| rs150266910 | 1:23442265 | T | 0.1731 | 0.0267 | 1.00 | 0.178 | 9.57E-11 |
| rs6429422 | 1:243472801 | T | -0.246 | 0.0185 | 0.97 | 0.322 | 3.29E-40 |
| rs6686889 | 1:25030470 | T | 0.1918 | 0.0199 | 0.96 | 0.253 | 6.95E-22 |
| rs3737801 | 1:27960832 | C | 0.3886 | 0.0601 | 0.98 | 0.072 | 1.02E-10 |
| rs2493292 | 1:3328659 | T | 0.2481 | 0.0251 | 0.97 | 0.144 | 5.54E-23 |
| rs4360494 | 1:38455891 | C | 0.2978 | 0.0215 | 0.87 | 0.449 | 1.29E-43 |
| rs11210029 | 1:41865293 | A | -0.203 | 0.0313 | 0.99 | 0.362 | 8.92E-11 |
| rs7515635 | 1:42408070 | T | 0.2509 | 0.0303 | 0.94 | 0.462 | 1.25E-16 |
| rs4926923 | 1:48109225 | T | 0.1918 | 0.0308 | 0.98 | 0.089 | 4.75E-10 |
| rs11579440 | 1:49052423 | T | 0.2674 | 0.0425 | 0.99 | 0.151 | 3.24E-10 |
| rs147696085 | 1:51021867 | A | -0.2296 | 0.0357 | 0.97 | 0.094 | 1.33E-10 |
| rs112557609 | 1:56576924 | A | 0.2164 | 0.0216 | 0.99 | 0.343 | 1.50E-23 |
| rs60199046 | 1:59663341 | A | 0.3472 | 0.0225 | 0.99 | 0.289 | 1.18E-53 |
| rs709209 | 1:6278414 | A | 0.1238 | 0.0223 | 1.00 | 0.342 | 2.82E-08 |
| rs4908678 | 1:7739250 | T | -0.1124 | 0.018 | 0.99 | 0.371 | 4.59E-10 |
| rs2252865 | 1:8422676 | T | 0.1189 | 0.0181 | 1.00 | 0.354 | 5.51E-11 |
| rs10923038 | 1:88651771 | A | 0.2008 | 0.0313 | 0.99 | 0.381 | 1.36E-10 |
| rs10922502 | 1:89360158 | A | -0.2863 | 0.0313 | 0.99 | 0.372 | 6.14E-20 |
| rs2065152 | 1:90228519 | T | 0.1103 | 0.018 | 1.00 | 0.356 | 8.95E-10 |
| rs17516329 | 1:92319781 | A | 0.1417 | 0.0221 | 0.99 | 0.310 | 1.58E-10 |
| rs9662255 | 1:9441949 | A | -0.2167 | 0.021 | 0.98 | 0.436 | 6.90E-25 |
| rs150194832 | 2:106126880 | C | -0.2147 | 0.0354 | 1.00 | 0.091 | 1.35E-09 |
| rs62158170 | 2:114082175 | A | 0.1645 | 0.0211 | 0.99 | 0.216 | 6.63E-15 |
| rs10864859 | 2:121440218 | T | 0.1962 | 0.0325 | 1.00 | 0.079 | 1.52E-09 |
| rs4954192 | 2:135632981 | T | -0.1225 | 0.0179 | 1.00 | 0.372 | 8.15E-12 |
| rs1438896 | 2:145646072 | T | 0.195 | 0.0189 | 0.99 | 0.298 | 4.56E-25 |
| rs62169544 | 2:146950908 | A | -0.1207 | 0.0175 | 0.99 | 0.446 | 4.96E-12 |
| rs12990959 | 2:148572160 | T | -0.1271 | 0.0187 | 1.00 | 0.308 | 1.11E-11 |
| rs3175 | 2:153618773 | A | 0.1287 | 0.022 | 0.96 | 0.349 | 4.63E-09 |
| rs55732192 | 2:162278233 | T | -0.3358 | 0.0521 | 0.99 | 0.094 | 1.15E-10 |
| rs1446468 | 2:164963486 | T | -0.5063 | 0.0304 | 0.98 | 0.454 | 4.38E-62 |
| rs6712203 | 2:165557318 | T | -0.2092 | 0.0313 | 0.99 | 0.378 | 2.41E-11 |
| rs6758859 | 2:173965056 | T | 0.1211 | 0.0179 | 1.00 | 0.363 | 1.45E-11 |
| rs11694601 | 2:174949358 | A | -0.1909 | 0.0309 | 0.98 | 0.400 | 6.41E-10 |
| rs72914576 | 2:175529967 | C | -0.2081 | 0.0263 | 1.00 | 0.183 | 2.72E-15 |
| rs60148403 | 2:177989414 | A | -0.1674 | 0.0261 | 0.99 | 0.198 | 1.38E-10 |
| rs1837164 | 2:178716601 | A | 0.1824 | 0.0311 | 1.00 | 0.365 | 4.66E-09 |
| rs79146658 | 2:179786068 | T | -0.3344 | 0.0312 | 0.98 | 0.086 | 7.85E-27 |
| rs10184839 | 2:181946115 | A | -0.1398 | 0.019 | 1.00 | 0.296 | 2.09E-13 |
| rs16823124 | 2:183224127 | A | 0.2276 | 0.0187 | 0.92 | 0.307 | 4.06E-34 |
| rs7592578 | 2:191439591 | T | -0.1998 | 0.0224 | 1.00 | 0.194 | 4.71E-19 |
| rs1344653 | 2:19730845 | A | -0.2534 | 0.03 | 0.98 | 0.500 | 3.09E-17 |
| rs296797 | 2:201102905 | T | 0.2161 | 0.0308 | 0.99 | 0.398 | 2.16E-12 |
| rs1469760 | 2:204125426 | T | -0.1891 | 0.0208 | 1.00 | 0.415 | 1.16E-19 |
| rs2162003 | 2:205077128 | T | 0.1279 | 0.0183 | 0.97 | 0.391 | 3.20E-12 |
| rs1263671 | 2:207996447 | T | -0.1394 | 0.0238 | 0.98 | 0.164 | 4.69E-09 |
| rs7255 | 2:20878820 | T | -0.192 | 0.0207 | 1.00 | 0.454 | 1.70E-20 |
| rs1047891 | 2:211540507 | A | -0.2528 | 0.0328 | 1.00 | 0.316 | 1.37E-14 |
| rs66774912 | 2:21423532 | A | -0.1826 | 0.0299 | 0.99 | 0.135 | 1.06E-09 |
| rs1250259 | 2:216300482 | A | -0.2782 | 0.0233 | 0.99 | 0.262 | 8.61E-33 |
| rs4674114 | 2:217659266 | A | -0.2116 | 0.0256 | 1.00 | 0.202 | 1.28E-16 |
| rs1063281 | 2:218668732 | T | -0.1623 | 0.0179 | 0.98 | 0.397 | 1.21E-19 |
| rs2972146 | 2:227100698 | T | 0.2532 | 0.0313 | 0.92 | 0.358 | 6.53E-16 |
| rs12052878 | 2:238227594 | A | -0.1497 | 0.022 | 1.00 | 0.316 | 1.11E-11 |
| rs10779936 | 2:23950200 | A | -0.1526 | 0.0226 | 1.00 | 0.285 | 1.41E-11 |
| rs4507125 | 2:239864732 | A | -0.1244 | 0.0211 | 1.00 | 0.208 | 3.60E-09 |
| rs55701159 | 2:25139596 | T | 0.3959 | 0.0481 | 0.99 | 0.113 | 1.73E-16 |
| rs1275988 | 2:26914364 | T | -0.541 | 0.0308 | 0.99 | 0.389 | 4.42E-69 |
| rs9678851 | 2:27887034 | A | -0.1722 | 0.0307 | 1.00 | 0.433 | 1.99E-08 |
| rs7562 | 2:28635740 | T | 0.2313 | 0.0305 | 0.98 | 0.479 | 3.26E-14 |
| rs13420463 | 2:37517566 | A | 0.3143 | 0.036 | 1.00 | 0.227 | 2.72E-18 |
| rs4952611 | 2:40567743 | T | -0.1401 | 0.018 | 0.99 | 0.421 | 7.38E-15 |
| rs11681462 | 2:42352567 | A | -0.1325 | 0.0214 | 0.99 | 0.215 | 5.96E-10 |
| rs76326501 | 2:43167878 | A | 0.3618 | 0.0305 | 0.98 | 0.091 | 2.17E-32 |
| rs11690961 | 2:46363336 | A | 0.3036 | 0.0319 | 0.99 | 0.117 | 1.93E-21 |
| rs10189186 | 2:53025757 | A | 0.1893 | 0.0302 | 0.98 | 0.474 | 3.91E-10 |
| rs1975487 | 2:55809054 | A | -0.141 | 0.0176 | 0.94 | 0.481 | 1.01E-15 |
| rs925484 | 2:60611437 | C | -0.1492 | 0.0209 | 1.00 | 0.405 | 8.66E-13 |
| rs7608483 | 2:61836235 | A | 0.1171 | 0.0176 | 1.00 | 0.413 | 2.83E-11 |
| rs13014371 | 2:64217786 | T | -0.1176 | 0.0175 | 0.99 | 0.430 | 1.68E-11 |
| rs2540951 | 2:65276736 | A | 0.2243 | 0.021 | 1.00 | 0.380 | 1.26E-26 |
| rs12052761 | 2:69065841 | A | -0.1229 | 0.0177 | 0.99 | 0.394 | 4.22E-12 |
| rs3771371 | 2:71627539 | T | -0.133 | 0.0206 | 0.99 | 0.430 | 1.15E-10 |
| rs10193543 | 2:72483329 | T | 0.1391 | 0.0235 | 1.00 | 0.167 | 3.20E-09 |
| rs1876487 | 2:73114352 | A | -0.1099 | 0.0196 | 0.99 | 0.295 | 1.93E-08 |
| rs11689667 | 2:85491365 | T | 0.2029 | 0.0206 | 1.00 | 0.456 | 7.36E-23 |
| rs72847885 | 2:86326717 | A | 0.2413 | 0.0318 | 1.00 | 0.338 | 3.08E-14 |
| rs2175337 | 2:9298590 | A | 0.1656 | 0.021 | 0.99 | 0.384 | 3.52E-15 |
| rs2579519 | 2:96675166 | T | -0.1818 | 0.018 | 1.00 | 0.383 | 4.24E-24 |
| rs28675079 | 3:111500002 | A | -0.1444 | 0.0222 | 1.00 | 0.182 | 8.34E-11 |
| rs347591 | 3:11290122 | T | 0.3181 | 0.032 | 0.97 | 0.338 | 2.87E-23 |
| rs6806529 | 3:123049938 | A | 0.1372 | 0.0209 | 0.99 | 0.437 | 5.81E-11 |
| rs6438857 | 3:124557643 | T | 0.2736 | 0.0305 | 0.99 | 0.420 | 3.13E-19 |
| rs62270945 | 3:128201889 | T | 0.5276 | 0.0651 | 1.00 | 0.029 | 5.17E-16 |
| rs2306374 | 3:138119952 | T | -0.1774 | 0.0236 | 0.98 | 0.162 | 5.24E-14 |
| rs729639 | 3:13826854 | T | -0.1246 | 0.0216 | 0.99 | 0.340 | 8.35E-09 |
| rs16851397 | 3:141134818 | A | -0.3942 | 0.0415 | 0.99 | 0.047 | 2.04E-21 |
| rs62278541 | 3:142631909 | A | 0.1677 | 0.0214 | 0.99 | 0.353 | 4.84E-15 |
| rs11128722 | 3:14958126 | A | -0.2865 | 0.0309 | 0.93 | 0.430 | 1.93E-20 |
| rs73158427 | 3:153721493 | A | 0.1801 | 0.0235 | 1.00 | 0.162 | 1.76E-14 |
| rs143112823 | 3:154707967 | A | -0.4171 | 0.0557 | 0.99 | 0.088 | 7.16E-14 |
| rs419076 | 3:169100886 | T | 0.2755 | 0.0173 | 0.97 | 0.473 | 2.66E-57 |
| rs4894535 | 3:171995605 | T | 0.1848 | 0.0281 | 0.99 | 0.158 | 4.72E-11 |
| rs73171158 | 3:176927949 | T | -0.1288 | 0.0217 | 0.92 | 0.463 | 2.82E-09 |
| rs7611674 | 3:179169230 | T | 0.1576 | 0.0223 | 0.98 | 0.191 | 1.67E-12 |
| rs262986 | 3:183435713 | A | -0.2371 | 0.0305 | 0.99 | 0.470 | 7.67E-15 |
| rs12374077 | 3:185317674 | C | 0.1748 | 0.0182 | 0.99 | 0.344 | 8.05E-22 |
| rs13082711 | 3:27537909 | T | -0.1778 | 0.0203 | 0.93 | 0.239 | 1.70E-18 |
| rs72851229 | 3:29374219 | C | -0.1364 | 0.0231 | 0.97 | 0.175 | 3.63E-09 |
| rs9815354 | 3:41912651 | A | -0.5267 | 0.028 | 0.98 | 0.164 | 6.22E-79 |
| rs6797587 | 3:48197614 | A | -0.2378 | 0.0185 | 0.96 | 0.329 | 6.56E-38 |
| rs36022378 | 3:49913705 | T | -0.1765 | 0.0219 | 1.00 | 0.200 | 8.60E-16 |
| rs13303 | 3:52558008 | T | -0.1326 | 0.0208 | 0.99 | 0.437 | 1.75E-10 |
| rs9810888 | 3:53635595 | T | -0.1151 | 0.0175 | 1.00 | 0.498 | 4.38E-11 |
| rs9827472 | 3:56726646 | T | -0.133 | 0.0181 | 1.00 | 0.365 | 1.75E-13 |
| rs12486605 | 3:57706503 | T | -0.1508 | 0.0176 | 1.00 | 0.428 | 1.01E-17 |
| rs3774702 | 3:63856870 | A | 0.147 | 0.0228 | 0.99 | 0.178 | 1.18E-10 |
| rs918466 | 3:64710253 | A | -0.1402 | 0.0177 | 0.99 | 0.410 | 2.67E-15 |
| rs7630745 | 3:66427029 | T | 0.1636 | 0.0215 | 1.00 | 0.342 | 2.73E-14 |
| rs4499560 | 3:70920485 | A | -0.2199 | 0.0326 | 0.98 | 0.314 | 1.46E-11 |
| rs729448 | 3:73260545 | A | -0.1303 | 0.0207 | 0.99 | 0.450 | 2.76E-10 |
| rs1375564 | 3:85656311 | T | 0.2579 | 0.0315 | 0.98 | 0.362 | 2.84E-16 |
| rs13107325 | 4:103188709 | T | -0.6747 | 0.0339 | 1.00 | 0.074 | 3.72E-88 |
| rs4699165 | 4:106109381 | A | 0.1377 | 0.0214 | 1.00 | 0.363 | 1.16E-10 |
| rs13112725 | 4:106911742 | C | 0.4137 | 0.0358 | 0.99 | 0.237 | 6.81E-31 |
| rs7694643 | 4:109017528 | A | -0.1318 | 0.0181 | 0.99 | 0.355 | 3.07E-13 |
| rs6825911 | 4:111381638 | T | -0.202 | 0.0215 | 1.00 | 0.208 | 6.94E-21 |
| rs4834735 | 4:119958809 | T | 0.1511 | 0.0254 | 0.99 | 0.136 | 2.64E-09 |
| rs66887589 | 4:120509279 | T | -0.161 | 0.0174 | 0.99 | 0.478 | 1.83E-20 |
| rs3097937 | 4:124794644 | A | 0.2226 | 0.0381 | 0.99 | 0.201 | 4.95E-09 |
| rs1250129 | 4:1254930 | A | -0.2069 | 0.0322 | 1.00 | 0.116 | 1.29E-10 |
| rs72719160 | 4:144051276 | A | -0.2243 | 0.0324 | 1.00 | 0.315 | 4.34E-12 |
| rs4292285 | 4:145271954 | A | -0.1073 | 0.0177 | 0.98 | 0.402 | 1.28E-09 |
| rs4835266 | 4:146821725 | T | 0.1645 | 0.021 | 0.97 | 0.484 | 4.24E-15 |
| rs10305838 | 4:148400256 | T | -0.2542 | 0.0293 | 1.00 | 0.140 | 4.65E-18 |
| rs6823767 | 4:151295085 | T | -0.2129 | 0.0341 | 1.00 | 0.277 | 4.40E-10 |
| rs13139571 | 4:156645513 | A | -0.2408 | 0.0203 | 1.00 | 0.237 | 2.29E-32 |
| rs11730129 | 4:16032948 | T | -0.1608 | 0.0249 | 1.00 | 0.218 | 9.98E-11 |
| rs869396 | 4:169688000 | A | -0.2221 | 0.0207 | 0.99 | 0.467 | 8.00E-27 |
| rs2498323 | 4:3451109 | A | 0.2957 | 0.035 | 1.00 | 0.100 | 3.07E-17 |
| rs1878825 | 4:36091370 | C | -0.1072 | 0.0183 | 0.98 | 0.351 | 4.62E-09 |
| rs2291435 | 4:38387395 | T | -0.262 | 0.0303 | 0.98 | 0.467 | 5.31E-18 |
| rs871606 | 4:54799245 | T | 0.5261 | 0.0335 | 0.99 | 0.105 | 1.93E-55 |
| rs6551716 | 4:63575696 | A | 0.1478 | 0.0251 | 0.99 | 0.144 | 3.91E-09 |
| rs10008637 | 4:77414144 | T | 0.2157 | 0.0302 | 1.00 | 0.462 | 9.24E-13 |
| rs16998073 | 4:81184341 | A | -0.494 | 0.0192 | 1.00 | 0.291 | 3.74E-146 |
| rs2014912 | 4:86715670 | T | 0.4772 | 0.042 | 0.92 | 0.153 | 6.97E-30 |
| rs13149209 | 4:89750668 | T | 0.281 | 0.0367 | 1.00 | 0.215 | 1.97E-14 |
| rs79409628 | 5:108113740 | T | -0.3086 | 0.0368 | 1.00 | 0.084 | 5.24E-17 |
| rs9885577 | 5:121194226 | T | 0.1621 | 0.0218 | 0.98 | 0.367 | 1.01E-13 |
| rs13359291 | 5:122476457 | A | 0.4333 | 0.0415 | 0.99 | 0.157 | 1.64E-25 |
| rs6595838 | 5:127868199 | A | 0.3229 | 0.0331 | 1.00 | 0.299 | 1.54E-22 |
| rs12521868 | 5:131784393 | T | -0.1403 | 0.0176 | 0.91 | 0.423 | 1.83E-15 |
| rs2400509 | 5:147696018 | A | -0.1476 | 0.0234 | 1.00 | 0.257 | 2.69E-10 |
| rs9687065 | 5:148391140 | A | 0.2199 | 0.0222 | 0.99 | 0.190 | 4.85E-23 |
| rs157678 | 5:156145654 | A | -0.1457 | 0.0223 | 0.97 | 0.337 | 6.85E-11 |
| rs114503346 | 5:172192350 | T | -0.2678 | 0.0426 | 0.97 | 0.047 | 3.10E-10 |
| rs72812846 | 5:173377636 | A | -0.2053 | 0.0197 | 0.98 | 0.279 | 2.15E-25 |
| rs28362590 | 5:176731452 | T | 0.1242 | 0.0203 | 1.00 | 0.247 | 8.70E-10 |
| rs12153395 | 5:179411477 | A | -0.3303 | 0.0486 | 0.98 | 0.116 | 1.07E-11 |
| rs1173771 | 5:32815028 | A | -0.6321 | 0.0307 | 0.96 | 0.399 | 5.92E-94 |
| rs4957026 | 5:361148 | A | 0.1982 | 0.0323 | 1.00 | 0.332 | 8.12E-10 |
| rs1694068 | 5:53283630 | A | 0.2657 | 0.0311 | 1.00 | 0.382 | 1.18E-17 |
| rs1848510 | 5:57754005 | A | 0.1256 | 0.0181 | 1.00 | 0.365 | 4.10E-12 |
| rs6875372 | 5:64079015 | A | 0.1886 | 0.0303 | 0.98 | 0.486 | 4.80E-10 |
| rs72761109 | 5:71506529 | T | 0.1583 | 0.0223 | 1.00 | 0.310 | 1.19E-12 |
| rs10078021 | 5:75038431 | T | -0.1534 | 0.0182 | 0.99 | 0.372 | 3.15E-17 |
| rs10057188 | 5:77837789 | A | -0.1796 | 0.0208 | 0.97 | 0.459 | 5.79E-18 |
| rs10059921 | 5:87514515 | T | -0.4248 | 0.0587 | 0.99 | 0.083 | 4.39E-13 |
| rs62380354 | 5:89484911 | A | 0.1825 | 0.0291 | 0.96 | 0.111 | 3.68E-10 |
| rs1871190 | 5:97953719 | T | 0.1954 | 0.0324 | 0.99 | 0.326 | 1.66E-09 |
| rs72613227 | 6:106320771 | A | -0.1884 | 0.0285 | 0.99 | 0.130 | 3.87E-11 |
| rs9486916 | 6:109013930 | T | 0.1842 | 0.0261 | 0.96 | 0.194 | 1.84E-12 |
| rs3822857 | 6:116313931 | C | -0.1238 | 0.018 | 0.99 | 0.379 | 6.09E-12 |
| rs2693560 | 6:117523671 | A | -0.1501 | 0.0181 | 0.99 | 0.371 | 1.09E-16 |
| rs9372498 | 6:118572486 | A | 0.2731 | 0.0319 | 0.98 | 0.081 | 1.13E-17 |
| rs11154027 | 6:121781390 | T | 0.1439 | 0.0208 | 1.00 | 0.461 | 4.47E-12 |
| rs13209747 | 6:127115454 | T | 0.3017 | 0.0175 | 0.97 | 0.441 | 6.23E-67 |
| rs9349379 | 6:12903957 | A | 0.2677 | 0.0212 | 0.99 | 0.407 | 1.32E-36 |
| rs6941056 | 6:143591821 | C | 0.2008 | 0.0207 | 0.99 | 0.438 | 3.57E-22 |
| rs7765526 | 6:147713764 | A | 0.201 | 0.0307 | 0.98 | 0.460 | 5.88E-11 |
| rs17080102 | 6:151004770 | C | -0.4853 | 0.034 | 0.97 | 0.070 | 3.91E-46 |
| rs13192976 | 6:152312415 | A | -0.4634 | 0.0329 | 0.99 | 0.111 | 5.22E-45 |
| rs9479509 | 6:153427265 | A | -0.1152 | 0.019 | 1.00 | 0.288 | 1.21E-09 |
| rs449789 | 6:159699125 | C | 0.3721 | 0.0297 | 0.97 | 0.139 | 4.50E-36 |
| rs555754 | 6:160769423 | A | -0.1392 | 0.0205 | 1.00 | 0.469 | 1.04E-11 |
| rs9456648 | 6:161712235 | T | -0.1166 | 0.0185 | 1.00 | 0.327 | 2.76E-10 |
| rs11961593 | 6:166164137 | T | -0.3158 | 0.0349 | 0.97 | 0.070 | 1.49E-19 |
| rs1322639 | 6:169587103 | A | 0.2978 | 0.0247 | 1.00 | 0.224 | 2.41E-33 |
| rs12216497 | 6:19028623 | T | 0.1307 | 0.0206 | 0.99 | 0.439 | 2.25E-10 |
| rs6911827 | 6:22130601 | T | 0.2378 | 0.0306 | 0.97 | 0.458 | 7.96E-15 |
| rs1799945 | 6:26091179 | C | -0.3888 | 0.0244 | 0.93 | 0.150 | 3.88E-57 |
| rs926552 | 6:29548089 | A | -0.2501 | 0.0272 | 0.90 | 0.133 | 3.54E-20 |
| rs409558 | 6:31708147 | T | 0.336 | 0.0293 | 0.81 | 0.150 | 2.28E-30 |
| rs4714224 | 6:39186743 | C | -0.1358 | 0.0196 | 0.99 | 0.275 | 3.78E-12 |
| rs1563788 | 6:43308363 | T | 0.3385 | 0.0332 | 0.95 | 0.287 | 2.36E-24 |
| rs78648104 | 6:50683009 | T | -0.4287 | 0.0541 | 1.00 | 0.093 | 2.37E-15 |
| rs13205180 | 6:51832494 | T | 0.1721 | 0.0174 | 0.98 | 0.489 | 4.38E-23 |
| rs631441 | 6:53994626 | T | -0.1543 | 0.0222 | 1.00 | 0.310 | 3.56E-12 |
| rs1925153 | 6:56102780 | T | -0.1936 | 0.0213 | 0.99 | 0.445 | 8.81E-20 |
| rs504691 | 6:72206620 | A | -0.1177 | 0.0177 | 0.99 | 0.398 | 3.14E-11 |
| rs9392172 | 6:7723962 | C | -0.1845 | 0.0205 | 1.00 | 0.469 | 2.57E-19 |
| rs10943605 | 6:79655477 | A | 0.1723 | 0.0173 | 1.00 | 0.488 | 2.93E-23 |
| rs7753695 | 6:80818531 | T | 0.1031 | 0.0176 | 1.00 | 0.446 | 4.77E-09 |
| rs114275780 | 6:8224648 | A | 0.2577 | 0.0459 | 0.95 | 0.066 | 1.95E-08 |
| rs9449350 | 6:82281417 | T | -0.2189 | 0.0323 | 1.00 | 0.315 | 1.19E-11 |
| rs60255247 | 6:85283253 | A | 0.2491 | 0.033 | 0.98 | 0.114 | 4.53E-14 |
| rs35410524 | 6:96885405 | T | 0.3368 | 0.0387 | 0.98 | 0.189 | 3.19E-18 |
| rs12705090 | 7:100467700 | T | -0.2361 | 0.0262 | 1.00 | 0.188 | 2.17E-19 |
| rs17477177 | 7:106411858 | T | -0.7351 | 0.0375 | 0.99 | 0.204 | 9.01E-86 |
| rs1997571 | 7:116198621 | A | -0.1448 | 0.0208 | 1.00 | 0.409 | 3.46E-12 |
| rs4728142 | 7:128573967 | A | -0.1814 | 0.0305 | 0.91 | 0.444 | 2.59E-09 |
| rs11556924 | 7:129663496 | T | -0.181 | 0.0181 | 0.99 | 0.383 | 1.83E-23 |
| rs13238550 | 7:131059056 | A | 0.2572 | 0.0309 | 1.00 | 0.397 | 7.80E-17 |
| rs10267979 | 7:136618188 | A | -0.1317 | 0.0218 | 1.00 | 0.324 | 1.62E-09 |
| rs141212865 | 7:139404666 | A | 0.301 | 0.0387 | 0.98 | 0.190 | 7.68E-15 |
| rs13240040 | 7:14375977 | A | 0.1186 | 0.019 | 0.99 | 0.322 | 3.98E-10 |
| rs73727605 | 7:149474622 | A | 0.2853 | 0.0423 | 1.00 | 0.067 | 1.53E-11 |
| rs3918226 | 7:150690176 | T | 0.6117 | 0.0329 | 0.98 | 0.081 | 5.31E-77 |
| rs10224002 | 7:151415041 | A | -0.3672 | 0.0337 | 0.96 | 0.286 | 1.31E-27 |
| rs1870735 | 7:155744303 | C | 0.206 | 0.0311 | 0.98 | 0.452 | 3.61E-11 |
| rs2107595 | 7:19049388 | A | 0.4435 | 0.0282 | 1.00 | 0.159 | 8.24E-56 |
| rs6959688 | 7:1966831 | A | -0.2344 | 0.031 | 0.99 | 0.403 | 4.22E-14 |
| rs4507656 | 7:22156538 | C | -0.1487 | 0.0199 | 0.97 | 0.304 | 8.69E-14 |
| rs12979 | 7:24738164 | C | 0.2739 | 0.0449 | 0.98 | 0.134 | 1.09E-09 |
| rs2969070 | 7:2512545 | A | -0.1791 | 0.0179 | 0.93 | 0.369 | 1.76E-23 |
| rs1055144 | 7:25871109 | T | 0.152 | 0.0259 | 0.99 | 0.192 | 4.58E-09 |
| rs6969780 | 7:27159136 | C | 0.2957 | 0.0526 | 0.98 | 0.092 | 1.88E-08 |
| rs917275 | 7:28658522 | A | -0.1887 | 0.0212 | 1.00 | 0.390 | 5.02E-19 |
| rs342989 | 7:35467896 | A | 0.1631 | 0.0207 | 1.00 | 0.234 | 3.05E-15 |
| rs2052263 | 7:36225818 | A | 0.1983 | 0.0327 | 1.00 | 0.121 | 1.41E-09 |
| rs76206723 | 7:40447971 | A | -0.3632 | 0.0333 | 0.99 | 0.107 | 1.27E-27 |
| rs1004558 | 7:44240407 | T | 0.1793 | 0.027 | 1.00 | 0.179 | 3.14E-11 |
| rs73105827 | 7:45036785 | T | -0.1878 | 0.0313 | 0.97 | 0.090 | 2.07E-09 |
| rs11977526 | 7:46008110 | A | -0.4308 | 0.0211 | 0.96 | 0.402 | 1.75E-92 |
| rs12668436 | 7:47548893 | T | -0.1709 | 0.0238 | 1.00 | 0.248 | 6.47E-13 |
| rs17454517 | 7:50915776 | A | 0.1216 | 0.0174 | 1.00 | 0.498 | 2.65E-12 |
| rs1468520 | 7:7290732 | A | -0.1638 | 0.0234 | 0.99 | 0.162 | 2.49E-12 |
| rs1091811 | 7:73491212 | A | -0.1745 | 0.0275 | 0.999 | 0.170 | 2.28E-10 |
| rs10245696 | 7:90449362 | A | 0.1549 | 0.0209 | 1.00 | 0.400 | 1.18E-13 |
| rs2282978 | 7:92264410 | T | 0.2928 | 0.0218 | 0.98 | 0.332 | 3.46E-41 |
| rs2978098 | 8:101676675 | A | 0.1548 | 0.0176 | 0.99 | 0.454 | 1.33E-18 |
| rs142449193 | 8:102750597 | T | -0.4549 | 0.074 | 0.97 | 0.044 | 7.86E-10 |
| rs2513877 | 8:103883630 | A | -0.1294 | 0.022 | 1.00 | 0.197 | 4.22E-09 |
| rs35783704 | 8:105966258 | A | -0.4619 | 0.0507 | 0.98 | 0.104 | 8.81E-20 |
| rs2898290 | 8:11433909 | T | 0.3123 | 0.0304 | 0.95 | 0.476 | 1.08E-24 |
| rs2071518 | 8:120435812 | T | 0.4583 | 0.0233 | 0.99 | 0.261 | 2.94E-86 |
| rs62523863 | 8:126520544 | A | 0.2689 | 0.0368 | 0.99 | 0.219 | 2.87E-13 |
| rs894344 | 8:135612745 | A | -0.1267 | 0.0176 | 0.99 | 0.407 | 5.79E-13 |
| rs4454254 | 8:141060027 | A | -0.2467 | 0.0212 | 0.97 | 0.369 | 2.65E-31 |
| rs34591516 | 8:142367087 | T | 0.3121 | 0.0409 | 0.91 | 0.049 | 2.26E-14 |
| rs62524579 | 8:144060955 | A | -0.1656 | 0.0182 | 1.00 | 0.467 | 1.08E-19 |
| rs4875958 | 8:1721090 | A | 0.2256 | 0.0336 | 0.99 | 0.287 | 1.85E-11 |
| rs62503324 | 8:23400615 | T | 0.2033 | 0.0204 | 1.00 | 0.242 | 2.11E-23 |
| rs6557876 | 8:25900675 | T | -0.4156 | 0.0349 | 0.98 | 0.250 | 1.11E-32 |
| rs17321041 | 8:26445194 | T | 0.2313 | 0.0363 | 0.97 | 0.063 | 1.78E-10 |
| rs2979470 | 8:30288272 | T | 0.1991 | 0.0302 | 0.99 | 0.492 | 4.62E-11 |
| rs11991469 | 8:32413280 | C | -0.1327 | 0.0206 | 1.00 | 0.455 | 1.09E-10 |
| rs7845722 | 8:33309993 | A | -0.127 | 0.0209 | 1.00 | 0.396 | 1.25E-09 |
| rs2978456 | 8:42324765 | T | -0.1781 | 0.0212 | 0.99 | 0.449 | 5.14E-17 |
| rs4873492 | 8:51947549 | T | 0.3431 | 0.0403 | 0.99 | 0.172 | 1.61E-17 |
| rs2922895 | 8:6379932 | C | 0.1317 | 0.0175 | 1.00 | 0.431 | 5.86E-14 |
| rs2354862 | 8:64501744 | A | 0.2507 | 0.0317 | 0.99 | 0.362 | 2.42E-15 |
| rs13253358 | 8:68920135 | T | 0.2127 | 0.033 | 1.00 | 0.299 | 1.13E-10 |
| rs1350100 | 8:76054904 | A | 0.1653 | 0.0208 | 0.98 | 0.450 | 1.80E-15 |
| rs1449544 | 8:76591880 | A | 0.1927 | 0.0205 | 1.00 | 0.457 | 6.68E-21 |
| rs56345595 | 8:82814156 | A | 0.1329 | 0.0177 | 1.00 | 0.417 | 5.20E-14 |
| rs61040371 | 8:8503700 | T | 0.1836 | 0.0314 | 0.99 | 0.366 | 4.77E-09 |
| rs2142141 | 8:90940205 | C | -0.1054 | 0.0179 | 0.98 | 0.467 | 3.68E-09 |
| rs62526122 | 8:92769569 | A | 0.2108 | 0.0345 | 0.97 | 0.302 | 1.02E-09 |
| rs35287509 | 9:10594635 | T | -0.1082 | 0.0184 | 1.00 | 0.343 | 4.17E-09 |
| rs7043304 | 9:112358150 | T | 0.176 | 0.0249 | 0.99 | 0.143 | 1.63E-12 |
| rs111245230 | 9:113169775 | T | -0.7486 | 0.0834 | 0.97 | 0.035 | 2.81E-19 |
| rs13290326 | 9:116696625 | T | -0.1562 | 0.0204 | 0.99 | 0.496 | 2.11E-14 |
| rs1861881 | 9:119312256 | T | 0.115 | 0.0186 | 0.99 | 0.316 | 6.49E-10 |
| rs1953126 | 9:123640500 | T | 0.162 | 0.0215 | 0.95 | 0.354 | 4.34E-14 |
| rs10818775 | 9:125755571 | T | -0.2745 | 0.0315 | 0.97 | 0.121 | 2.73E-18 |
| rs72765298 | 9:127900996 | T | -0.3421 | 0.0315 | 0.98 | 0.125 | 1.56E-27 |
| rs6271 | 9:136522274 | T | -0.4313 | 0.0352 | 0.98 | 0.074 | 1.72E-34 |
| rs11145807 | 9:139520789 | A | 0.155 | 0.0184 | 0.98 | 0.408 | 4.10E-17 |
| rs520015 | 9:211762 | C | 0.2003 | 0.0301 | 1.00 | 0.488 | 2.84E-11 |
| rs4364717 | 9:21801530 | A | -0.1006 | 0.0174 | 1.00 | 0.453 | 7.56E-09 |
| rs9886665 | 9:22942770 | T | 0.2048 | 0.0343 | 0.98 | 0.263 | 2.47E-09 |
| rs12216886 | 9:2493751 | T | 0.1292 | 0.0221 | 0.99 | 0.190 | 4.76E-09 |
| rs4553000 | 9:34223553 | T | -0.1464 | 0.0204 | 1.00 | 0.486 | 7.47E-13 |
| rs76452347 | 9:35906471 | T | -0.2246 | 0.0229 | 0.99 | 0.205 | 9.37E-23 |
| rs60191654 | 9:753648 | A | -0.2382 | 0.0385 | 0.99 | 0.190 | 5.88E-10 |
| rs11141731 | 9:89888472 | T | -0.1258 | 0.0207 | 0.99 | 0.233 | 1.31E-09 |
| rs112184198 | 10:102604514 | A | -0.6626 | 0.0498 | 0.97 | 0.105 | 1.94E-40 |
| rs72847884 | 10:103115345 | A | 0.2664 | 0.0423 | 1.00 | 0.046 | 3.04E-10 |
| rs11191156 | 10:103702763 | A | -0.1573 | 0.0215 | 1.00 | 0.351 | 2.72E-13 |
| rs11191548 | 10:104846178 | T | 1.0983 | 0.0553 | 0.97 | 0.081 | 1.16E-87 |
| rs4387287 | 10:105677897 | A | 0.1575 | 0.0234 | 0.91 | 0.164 | 1.77E-11 |
| rs111777102 | 10:111965826 | T | 0.214 | 0.0354 | 0.99 | 0.064 | 1.56E-09 |
| rs34872471 | 10:114754071 | T | -0.2021 | 0.0226 | 1.00 | 0.292 | 4.16E-19 |
| rs1801253 | 10:115805056 | C | 0.3183 | 0.0197 | 0.99 | 0.266 | 1.57E-58 |
| rs72842207 | 10:121433675 | T | -0.2112 | 0.0211 | 1.00 | 0.215 | 1.10E-23 |
| rs11592107 | 10:122968964 | A | 0.3024 | 0.0326 | 1.00 | 0.310 | 1.55E-20 |
| rs72834453 | 10:124235226 | T | -0.3246 | 0.0465 | 0.98 | 0.123 | 2.95E-12 |
| rs1813353 | 10:18707448 | T | 0.3013 | 0.0183 | 0.96 | 0.337 | 5.97E-61 |
| rs72795925 | 10:20531420 | T | 0.1623 | 0.0247 | 0.99 | 0.222 | 5.13E-11 |
| rs10732433 | 10:21037294 | T | 0.1899 | 0.0208 | 0.98 | 0.423 | 5.77E-20 |
| rs3802517 | 10:28233469 | A | 0.2527 | 0.0301 | 1.00 | 0.468 | 4.65E-17 |
| rs1265842 | 10:28924901 | T | 0.1113 | 0.0174 | 0.99 | 0.480 | 1.70E-10 |
| rs9337951 | 10:30317073 | A | 0.2583 | 0.0227 | 0.99 | 0.342 | 4.24E-30 |
| rs11008355 | 10:31412561 | C | -0.1441 | 0.0241 | 0.99 | 0.239 | 2.10E-09 |
| rs10826995 | 10:32082658 | T | -0.1653 | 0.0228 | 0.98 | 0.285 | 3.80E-13 |
| rs76164690 | 10:32590362 | T | -0.154 | 0.025 | 0.99 | 0.143 | 7.19E-10 |
| rs2246438 | 10:45273079 | A | -0.1119 | 0.0194 | 1.00 | 0.276 | 7.81E-09 |
| rs10761530 | 10:62390726 | T | 0.117 | 0.0172 | 1.00 | 0.496 | 1.14E-11 |
| rs4590817 | 10:63467553 | C | -0.4151 | 0.0232 | 0.94 | 0.167 | 9.50E-72 |
| rs10995311 | 10:64564934 | C | 0.2017 | 0.0175 | 0.991 | 0.441 | 7.14E-31 |
| rs7090758 | 10:65335315 | T | -0.1533 | 0.0173 | 1.00 | 0.474 | 6.89E-19 |
| rs10823136 | 10:69855363 | T | -0.254 | 0.0397 | 0.97 | 0.069 | 1.58E-10 |
| rs10998362 | 10:70404159 | T | 0.1367 | 0.0228 | 0.98 | 0.313 | 2.13E-09 |
| rs77413490 | 10:89681688 | T | 0.4489 | 0.0764 | 0.99 | 0.043 | 4.27E-09 |
| rs11187142 | 10:94468685 | T | 0.3312 | 0.0496 | 0.99 | 0.105 | 2.53E-11 |
| rs932764 | 10:95895940 | A | -0.4303 | 0.0306 | 0.98 | 0.431 | 5.43E-45 |
| rs4494250 | 10:96563757 | A | 0.1917 | 0.0181 | 0.85 | 0.363 | 3.06E-26 |
| rs633185 | 11:100593538 | C | 0.376 | 0.0192 | 0.89 | 0.287 | 2.29E-85 |
| rs7129220 | 11:10350538 | A | 0.5002 | 0.0472 | 0.95 | 0.119 | 2.96E-26 |
| rs4754196 | 11:107096777 | A | -0.3486 | 0.0303 | 1.00 | 0.483 | 1.46E-30 |
| rs1076485 | 11:116772441 | T | 0.3388 | 0.0457 | 0.99 | 0.125 | 1.19E-13 |
| rs8258 | 11:117283676 | T | 0.2225 | 0.0212 | 0.99 | 0.374 | 9.42E-26 |
| rs12574332 | 11:122521123 | T | 0.2072 | 0.0266 | 1.00 | 0.120 | 6.14E-15 |
| rs11222084 | 11:130273230 | A | -0.4972 | 0.0214 | 1.00 | 0.362 | 5.19E-119 |
| rs900145 | 11:13293905 | T | 0.1493 | 0.0189 | 0.96 | 0.296 | 2.62E-15 |
| rs4757391 | 11:16302939 | T | -0.3041 | 0.0215 | 0.99 | 0.202 | 1.69E-45 |
| rs757081 | 11:17351683 | C | -0.3429 | 0.032 | 0.99 | 0.335 | 7.72E-27 |
| rs661348 | 11:1905292 | T | -0.4451 | 0.0316 | 1.00 | 0.425 | 5.23E-45 |
| rs10766533 | 11:19224677 | A | 0.2099 | 0.0337 | 0.99 | 0.281 | 4.69E-10 |
| rs11030119 | 11:27728102 | A | -0.1679 | 0.0189 | 0.91 | 0.302 | 7.31E-19 |
| rs871004 | 11:28512458 | A | 0.2336 | 0.0317 | 0.99 | 0.343 | 1.65E-13 |
| rs919045 | 11:31111810 | T | 0.1193 | 0.0179 | 1.00 | 0.367 | 2.75E-11 |
| rs4922591 | 11:32374199 | T | -0.1513 | 0.0214 | 0.97 | 0.377 | 1.39E-12 |
| rs4755947 | 11:45243463 | T | 0.2561 | 0.0313 | 0.98 | 0.120 | 2.75E-16 |
| rs7103648 | 11:47461783 | A | -0.235 | 0.0178 | 0.94 | 0.387 | 7.18E-40 |
| rs9667596 | 11:48691193 | T | 0.505 | 0.045 | 0.99 | 0.140 | 3.12E-29 |
| rs75905900 | 11:55113534 | A | 0.4174 | 0.0449 | 1.00 | 0.132 | 1.36E-20 |
| rs11607056 | 11:57496820 | T | -0.1829 | 0.0218 | 1.00 | 0.328 | 4.77E-17 |
| rs11229457 | 11:58207203 | T | -0.317 | 0.0369 | 0.98 | 0.213 | 8.45E-18 |
| rs751984 | 11:61278246 | T | 0.3937 | 0.0275 | 0.99 | 0.117 | 1.38E-46 |
| rs3741378 | 11:65408937 | T | -0.4087 | 0.0446 | 0.94 | 0.135 | 4.83E-20 |
| rs67330701 | 11:69079707 | T | -0.2798 | 0.0322 | 0.90 | 0.093 | 3.22E-18 |
| rs504217 | 11:72006086 | T | 0.2745 | 0.0335 | 1.00 | 0.072 | 2.51E-16 |
| rs2298807 | 11:73068571 | T | 0.1233 | 0.0211 | 1.00 | 0.210 | 4.89E-09 |
| rs7927515 | 11:76125330 | A | 0.2271 | 0.0319 | 0.97 | 0.346 | 1.05E-12 |
| rs59986178 | 11:77359909 | C | 0.176 | 0.0299 | 1.00 | 0.105 | 3.92E-09 |
| rs2450128 | 11:77940075 | A | -0.1505 | 0.024 | 1.00 | 0.152 | 3.53E-10 |
| rs110419 | 11:8252853 | A | 0.112 | 0.0171 | 0.84 | 0.478 | 6.16E-11 |
| rs7126805 | 11:828916 | A | 0.1511 | 0.0236 | 0.73 | 0.273 | 1.62E-10 |
| rs2289125 | 11:89224453 | A | -0.3847 | 0.0255 | 0.98 | 0.220 | 1.82E-51 |
| rs11021221 | 11:95308854 | A | -0.1877 | 0.0233 | 0.99 | 0.170 | 6.93E-16 |
| rs12184466 | 12:111281636 | T | 0.2533 | 0.0249 | 0.81 | 0.200 | 2.83E-24 |
| rs3184504 | 12:111884608 | T | 0.4999 | 0.0175 | 0.99 | 0.481 | 8.04E-180 |
| rs35444 | 12:115552437 | A | 0.2671 | 0.0179 | 1.00 | 0.386 | 1.91E-50 |
| rs11067763 | 12:116198341 | A | 0.2177 | 0.0288 | 1.00 | 0.101 | 4.43E-14 |
| rs1060105 | 12:123806219 | T | -0.1894 | 0.0216 | 0.81 | 0.203 | 2.11E-18 |
| rs117206641 | 12:133086888 | T | 0.3154 | 0.0499 | 0.99 | 0.140 | 2.66E-10 |
| rs28621435 | 12:13860990 | A | -0.2976 | 0.0482 | 0.97 | 0.112 | 6.47E-10 |
| rs61912333 | 12:19554817 | C | 0.1191 | 0.0176 | 0.98 | 0.492 | 1.13E-11 |
| rs12579720 | 12:20173764 | C | -0.2865 | 0.0203 | 1.00 | 0.244 | 3.46E-45 |
| rs17287293 | 12:24770878 | A | -0.197 | 0.0286 | 1.00 | 0.150 | 5.97E-12 |
| rs55935819 | 12:2521579 | A | 0.1271 | 0.0181 | 0.99 | 0.365 | 1.96E-12 |
| rs6487543 | 12:26438189 | A | 0.1325 | 0.0212 | 0.97 | 0.230 | 4.21E-10 |
| rs7965392 | 12:42540280 | A | 0.1118 | 0.0179 | 1.00 | 0.389 | 4.16E-10 |
| rs117233107 | 12:4328521 | A | -0.5334 | 0.0934 | 0.88 | 0.015 | 1.11E-08 |
| rs78998485 | 12:434755 | C | -0.2449 | 0.0346 | 1.00 | 0.258 | 1.48E-12 |
| rs11168245 | 12:48204499 | C | 0.1758 | 0.0205 | 1.00 | 0.239 | 1.09E-17 |
| rs1126930 | 12:49399132 | C | 0.6378 | 0.0856 | 1.00 | 0.035 | 9.48E-14 |
| rs7977389 | 12:49981722 | T | 0.1883 | 0.0334 | 0.99 | 0.106 | 1.70E-08 |
| rs7302981 | 12:50537815 | A | 0.2652 | 0.0177 | 0.99 | 0.378 | 1.69E-50 |
| rs61926181 | 12:50767037 | A | -0.4813 | 0.0484 | 0.98 | 0.040 | 2.76E-23 |
| rs73099903 | 12:53440779 | T | 0.4694 | 0.0557 | 1.00 | 0.082 | 3.43E-17 |
| rs7297416 | 12:54443090 | A | 0.3764 | 0.0329 | 0.88 | 0.305 | 2.90E-30 |
| rs4143175 | 12:67782397 | T | 0.2187 | 0.0352 | 0.99 | 0.242 | 5.10E-10 |
| rs521033 | 12:69951428 | A | -0.1802 | 0.0253 | 1.00 | 0.140 | 1.10E-12 |
| rs17249754 | 12:90060586 | A | -0.8446 | 0.0403 | 1.00 | 0.168 | 1.25E-97 |
| rs76785029 | 12:94882905 | T | -0.3473 | 0.0395 | 1.00 | 0.079 | 1.57E-18 |
| rs11108209 | 12:96109855 | T | -0.1901 | 0.03 | 0.99 | 0.094 | 2.40E-10 |
| rs3742182 | 13:111375132 | T | -0.1863 | 0.0261 | 1.00 | 0.186 | 9.00E-13 |
| rs9549328 | 13:113636156 | T | 0.2164 | 0.0247 | 0.98 | 0.230 | 1.77E-18 |
| rs7331680 | 13:115000650 | T | 0.4101 | 0.0423 | 1.00 | 0.149 | 3.35E-22 |
| rs2480171 | 13:21559858 | T | 0.2909 | 0.0467 | 1.00 | 0.113 | 4.69E-10 |
| rs55641580 | 13:25257917 | T | 0.1745 | 0.0265 | 1.00 | 0.124 | 4.79E-11 |
| rs1331012 | 13:27115424 | T | 0.2043 | 0.0338 | 1.00 | 0.271 | 1.49E-09 |
| rs9549297 | 13:41397482 | A | -0.1483 | 0.0229 | 0.99 | 0.178 | 9.27E-11 |
| rs75961402 | 13:56398286 | A | 0.2659 | 0.0418 | 1.00 | 0.155 | 1.95E-10 |
| rs3861113 | 13:72364382 | A | 0.2126 | 0.0322 | 0.99 | 0.076 | 3.95E-11 |
| rs1215469 | 13:80707408 | A | -0.1383 | 0.0211 | 0.99 | 0.229 | 5.23E-11 |
| rs1475130 | 14:100225144 | T | -0.1839 | 0.0216 | 1.00 | 0.346 | 1.89E-17 |
| rs11626434 | 14:101998443 | C | -0.1345 | 0.0219 | 0.99 | 0.359 | 7.87E-10 |
| rs34161718 | 14:104620193 | T | -0.1319 | 0.0216 | 0.97 | 0.241 | 1.07E-09 |
| rs452036 | 14:23865885 | A | -0.304 | 0.0215 | 1.00 | 0.355 | 1.96E-45 |
| rs8904 | 14:35871217 | A | 0.2104 | 0.0214 | 1.00 | 0.368 | 7.72E-23 |
| rs34983854 | 14:39858442 | A | -0.2056 | 0.0307 | 1.00 | 0.400 | 2.06E-11 |
| rs9888615 | 14:53377540 | T | -0.274 | 0.0332 | 0.92 | 0.291 | 1.46E-16 |
| rs731681 | 14:68010224 | C | -0.1071 | 0.0174 | 1.00 | 0.437 | 7.98E-10 |
| rs57786342 | 14:69260028 | A | 0.1423 | 0.0216 | 0.99 | 0.204 | 4.37E-11 |
| rs4903064 | 14:73279420 | T | 0.1543 | 0.0206 | 1.00 | 0.236 | 7.84E-14 |
| rs11627326 | 14:85785251 | C | 0.1546 | 0.0227 | 0.99 | 0.288 | 9.78E-12 |
| rs4904503 | 14:89565130 | T | 0.15 | 0.0225 | 0.99 | 0.298 | 2.52E-11 |
| rs11160085 | 14:93112102 | T | 0.1398 | 0.0228 | 0.99 | 0.304 | 8.61E-10 |
| rs9323988 | 14:98587630 | T | -0.1987 | 0.021 | 0.93 | 0.385 | 3.42E-21 |
| rs10873612 | 15:26105602 | T | -0.1096 | 0.0179 | 1.00 | 0.403 | 9.51E-10 |
| rs11629850 | 15:40317075 | A | 0.2297 | 0.0301 | 1.00 | 0.469 | 2.26E-14 |
| rs2925345 | 15:41311799 | T | 0.189 | 0.0174 | 1.00 | 0.470 | 1.60E-27 |
| rs4924570 | 15:41974660 | T | -0.1692 | 0.0181 | 0.99 | 0.370 | 9.62E-21 |
| rs1036477 | 15:48914926 | A | 0.4759 | 0.0336 | 1.00 | 0.103 | 1.77E-45 |
| rs3098186 | 15:50810621 | T | -0.1735 | 0.0207 | 0.99 | 0.480 | 4.40E-17 |
| rs956006 | 15:62808539 | T | -0.1619 | 0.022 | 1.00 | 0.331 | 1.84E-13 |
| rs7178615 | 15:66869072 | A | -0.1371 | 0.018 | 0.97 | 0.376 | 2.90E-14 |
| rs2289261 | 15:67457485 | C | -0.1485 | 0.0216 | 0.98 | 0.345 | 5.62E-12 |
| rs11853359 | 15:71621524 | A | -0.166 | 0.0183 | 1.00 | 0.333 | 1.30E-19 |
| rs61653296 | 15:74557817 | A | -0.1405 | 0.0218 | 1.00 | 0.198 | 1.13E-10 |
| rs1378942 | 15:75077367 | A | -0.388 | 0.0185 | 0.96 | 0.331 | 6.03E-98 |
| rs62011052 | 15:79156983 | T | -0.2574 | 0.0287 | 1.00 | 0.151 | 3.39E-19 |
| rs2759308 | 15:81016227 | A | 0.3155 | 0.0303 | 0.99 | 0.475 | 1.89E-25 |
| rs2034618 | 15:83799632 | T | -0.1157 | 0.0209 | 0.98 | 0.222 | 3.36E-08 |
| rs28611491 | 15:90641809 | T | 0.2544 | 0.0393 | 0.98 | 0.078 | 9.46E-11 |
| rs2521501 | 15:91437388 | A | -0.3693 | 0.0191 | 0.97 | 0.325 | 1.84E-83 |
| rs11632112 | 15:93468276 | C | -0.1645 | 0.0241 | 1.00 | 0.236 | 8.66E-12 |
| rs12906962 | 15:95312071 | T | -0.2378 | 0.0188 | 0.98 | 0.323 | 8.73E-37 |
| rs4984496 | 15:96635898 | T | 0.1763 | 0.0187 | 1.00 | 0.330 | 4.93E-21 |
| rs57327054 | 16:14487036 | T | -0.1173 | 0.0191 | 1.00 | 0.309 | 8.07E-10 |
| rs3915425 | 16:15912544 | T | 0.1913 | 0.022 | 0.99 | 0.318 | 4.00E-18 |
| rs13333226 | 16:20365654 | A | 0.2965 | 0.0223 | 0.98 | 0.184 | 2.94E-40 |
| rs28590346 | 16:2080653 | A | -0.1914 | 0.0191 | 1.00 | 0.342 | 9.84E-24 |
| rs11639856 | 16:24788645 | A | -0.2254 | 0.0258 | 0.99 | 0.193 | 2.65E-18 |
| rs72799341 | 16:30936743 | A | 0.1599 | 0.0204 | 0.85 | 0.240 | 4.92E-15 |
| rs2379829 | 16:3538873 | C | -0.2678 | 0.0342 | 1.00 | 0.268 | 4.48E-15 |
| rs4785955 | 16:4297651 | T | 0.1721 | 0.0256 | 0.98 | 0.213 | 1.79E-11 |
| rs12921187 | 16:4943019 | T | -0.175 | 0.0175 | 0.93 | 0.428 | 1.65E-23 |
| rs10468291 | 16:49768046 | A | -0.1166 | 0.0176 | 0.99 | 0.428 | 3.70E-11 |
| rs34941092 | 16:50550137 | A | -0.3225 | 0.0425 | 1.00 | 0.150 | 3.23E-14 |
| rs9932220 | 16:51758116 | A | -0.1591 | 0.021 | 1.00 | 0.217 | 3.76E-14 |
| rs28633979 | 16:65282820 | A | -0.2 | 0.0206 | 1.00 | 0.428 | 2.50E-22 |
| rs62047964 | 16:70729954 | T | 0.6572 | 0.0469 | 0.91 | 0.062 | 1.06E-44 |
| rs1012089 | 16:74171973 | C | -0.192 | 0.0302 | 0.99 | 0.475 | 1.95E-10 |
| rs35261357 | 16:75444572 | T | 0.2413 | 0.0209 | 1.00 | 0.415 | 1.01E-30 |
| rs56844452 | 16:80864776 | T | -0.3289 | 0.0406 | 0.99 | 0.070 | 5.44E-16 |
| rs8059962 | 16:81574197 | T | -0.1397 | 0.0177 | 0.42 | 0.421 | 3.38E-15 |
| rs7500448 | 16:83045790 | A | 0.3589 | 0.0239 | 0.98 | 0.254 | 3.62E-51 |
| rs3851018 | 16:86437811 | C | 0.1918 | 0.0309 | 0.98 | 0.433 | 5.40E-10 |
| rs6540125 | 16:87993889 | T | 0.2042 | 0.0317 | 0.99 | 0.334 | 1.21E-10 |
| rs1126464 | 16:89704365 | C | 0.2071 | 0.0208 | 1.00 | 0.243 | 1.89E-23 |
| rs12941318 | 17:1333598 | T | -0.1738 | 0.0211 | 0.94 | 0.496 | 1.60E-16 |
| rs4480845 | 17:1958609 | T | 0.3156 | 0.0316 | 0.98 | 0.359 | 1.85E-23 |
| rs9899540 | 17:30777924 | A | 0.2011 | 0.0316 | 1.00 | 0.384 | 1.87E-10 |
| rs7215084 | 17:3880148 | T | 0.1116 | 0.0173 | 1.00 | 0.486 | 1.17E-10 |
| rs79089478 | 17:40317241 | T | 0.3836 | 0.0643 | 0.99 | 0.027 | 2.38E-09 |
| rs12946454 | 17:43208121 | A | -0.4125 | 0.0341 | 0.86 | 0.265 | 1.01E-33 |
| rs17608766 | 17:45013271 | T | -0.5274 | 0.0295 | 0.90 | 0.144 | 2.12E-71 |
| rs7406910 | 17:46688256 | T | -0.4347 | 0.0533 | 1.00 | 0.088 | 3.39E-16 |
| rs12940887 | 17:47402807 | T | 0.2279 | 0.018 | 1.00 | 0.367 | 9.63E-37 |
| rs2645466 | 17:57853214 | A | -0.1358 | 0.0224 | 1.00 | 0.295 | 1.32E-09 |
| rs2240736 | 17:59485393 | T | 0.4088 | 0.0343 | 0.93 | 0.266 | 1.17E-32 |
| rs740698 | 17:60767151 | T | -0.2221 | 0.021 | 0.65 | 0.435 | 3.83E-26 |
| rs4308 | 17:61559625 | A | 0.1753 | 0.0181 | 0.73 | 0.377 | 3.74E-22 |
| rs6504213 | 17:62381714 | T | -0.2982 | 0.0312 | 0.99 | 0.408 | 1.25E-21 |
| rs112260610 | 17:64252393 | T | 0.2588 | 0.0435 | 0.99 | 0.144 | 2.69E-09 |
| rs4796514 | 17:6475090 | T | -0.2313 | 0.021 | 0.90 | 0.391 | 4.11E-28 |
| rs2467099 | 17:73949045 | T | -0.1428 | 0.0209 | 0.77 | 0.223 | 7.58E-12 |
| rs78378222 | 17:7571752 | T | 1.0488 | 0.0945 | 0.95 | 0.014 | 1.28E-28 |
| rs9302885 | 17:76799898 | A | 0.2242 | 0.0302 | 1.00 | 0.448 | 1.03E-13 |
| rs1154214 | 18:24546824 | T | -0.2031 | 0.0306 | 1.00 | 0.396 | 3.27E-11 |
| rs10164193 | 18:31161426 | T | -0.2196 | 0.0327 | 0.99 | 0.077 | 1.87E-11 |
| rs61735998 | 18:34289285 | T | 0.4712 | 0.0702 | 1.00 | 0.025 | 1.98E-11 |
| rs12958173 | 18:42141977 | A | 0.295 | 0.0329 | 0.95 | 0.297 | 2.97E-19 |
| rs7236548 | 18:43097750 | A | 0.3621 | 0.0264 | 0.99 | 0.185 | 8.48E-43 |
| rs36010659 | 18:48283949 | T | 0.2485 | 0.0294 | 0.98 | 0.141 | 2.98E-17 |
| rs72930904 | 18:52607301 | T | -0.14 | 0.0237 | 1.00 | 0.162 | 3.24E-09 |
| rs12605156 | 18:53498114 | A | 0.1418 | 0.0221 | 0.99 | 0.189 | 1.51E-10 |
| rs7235890 | 18:55732115 | T | -0.1692 | 0.0288 | 0.97 | 0.106 | 4.12E-09 |
| rs6567160 | 18:57829135 | T | 0.2242 | 0.0357 | 1.00 | 0.233 | 3.33E-10 |
| rs10460108 | 18:73034151 | A | 0.2141 | 0.0301 | 0.98 | 0.479 | 1.12E-12 |
| rs1047922 | 18:74070562 | T | -0.2121 | 0.0297 | 1.00 | 0.155 | 9.70E-13 |
| rs10409243 | 19:10332988 | T | -0.3077 | 0.0313 | 0.97 | 0.398 | 8.10E-23 |
| rs1529744 | 19:10841472 | T | -0.1209 | 0.022 | 0.99 | 0.322 | 3.87E-08 |
| rs167479 | 19:11526765 | T | -0.362 | 0.0188 | 1.00 | 0.472 | 1.67E-82 |
| rs3760994 | 19:1435771 | A | -0.144 | 0.0218 | 1.00 | 0.497 | 4.27E-11 |
| rs10418305 | 19:15278808 | C | -0.2849 | 0.0344 | 0.98 | 0.099 | 1.21E-16 |
| rs3745318 | 19:16436262 | T | 0.1396 | 0.0206 | 0.98 | 0.252 | 1.30E-11 |
| rs1077795 | 19:17222584 | A | 0.1987 | 0.0199 | 0.99 | 0.263 | 1.62E-23 |
| rs8111708 | 19:18558876 | A | 0.1515 | 0.0216 | 0.99 | 0.348 | 2.23E-12 |
| rs2304130 | 19:19789528 | A | -0.2396 | 0.0318 | 0.98 | 0.084 | 4.48E-14 |
| rs6511291 | 19:21950402 | T | -0.1158 | 0.0177 | 0.99 | 0.432 | 6.89E-11 |
| rs740406 | 19:2232221 | A | -0.5158 | 0.045 | 1.00 | 0.060 | 2.10E-30 |
| rs62104477 | 19:30294991 | T | 0.1703 | 0.0185 | 0.99 | 0.330 | 4.22E-20 |
| rs8105753 | 19:31927547 | A | 0.2178 | 0.0318 | 0.80 | 0.369 | 6.84E-12 |
| rs7256564 | 19:33889593 | A | 0.1955 | 0.0324 | 1.00 | 0.311 | 1.53E-09 |
| rs12983238 | 19:39438532 | A | -0.1266 | 0.0201 | 0.95 | 0.310 | 3.23E-10 |
| rs4803327 | 19:40840739 | T | 0.1149 | 0.0173 | 0.80 | 0.473 | 3.46E-11 |
| rs1800470 | 19:41858921 | A | -0.15 | 0.0213 | 1.00 | 0.370 | 1.76E-12 |
| rs73046792 | 19:49605705 | A | -0.3554 | 0.0426 | 1.00 | 0.163 | 7.23E-17 |
| rs4247374 | 19:7252756 | T | -0.6354 | 0.0456 | 1.00 | 0.139 | 4.52E-44 |
| rs2009733 | 19:8398714 | A | 0.1217 | 0.0176 | 1.00 | 0.495 | 5.10E-12 |
| rs1327235 | 20:10969030 | A | -0.3018 | 0.0173 | 1.00 | 0.471 | 4.76E-68 |
| rs2618647 | 20:17882452 | A | -0.1216 | 0.0174 | 0.99 | 0.485 | 2.70E-12 |
| rs6081613 | 20:19465907 | A | 0.2842 | 0.0229 | 0.97 | 0.275 | 2.55E-35 |
| rs2143635 | 20:2793063 | T | -0.2304 | 0.035 | 0.96 | 0.102 | 4.79E-11 |
| rs6060114 | 20:30169673 | T | 0.1689 | 0.0238 | 0.99 | 0.158 | 1.41E-12 |
| rs6141767 | 20:31225069 | C | 0.2147 | 0.0286 | 0.99 | 0.152 | 5.63E-14 |
| rs13042148 | 20:32298286 | T | -0.1674 | 0.0244 | 1.00 | 0.155 | 7.24E-12 |
| rs4810332 | 20:40268334 | A | -0.1715 | 0.0183 | 0.97 | 0.381 | 6.51E-21 |
| rs1764975 | 20:4101290 | A | 0.2819 | 0.0379 | 0.99 | 0.181 | 1.08E-13 |
| rs6031435 | 20:42797358 | A | -0.2592 | 0.0303 | 0.89 | 0.460 | 1.09E-17 |
| rs6095241 | 20:47308798 | A | -0.1358 | 0.0174 | 1.00 | 0.440 | 6.22E-15 |
| rs237485 | 20:48004238 | A | 0.1124 | 0.0191 | 0.99 | 0.306 | 3.63E-09 |
| rs6015450 | 20:57751117 | A | -0.4911 | 0.0266 | 0.97 | 0.124 | 5.37E-76 |
| rs35213536 | 20:62694319 | T | 0.2044 | 0.0205 | 1.00 | 0.243 | 2.54E-23 |
| rs6108168 | 20:8626271 | A | -0.1901 | 0.0199 | 0.97 | 0.255 | 1.10E-21 |
| rs1882961 | 21:16556367 | T | 0.2443 | 0.0326 | 0.98 | 0.306 | 6.69E-14 |
| rs11909120 | 21:30131872 | A | -0.1985 | 0.0293 | 1.00 | 0.145 | 1.24E-11 |
| rs11701033 | 21:33788341 | C | -0.2187 | 0.0392 | 1.00 | 0.182 | 2.52E-08 |
| rs12627651 | 21:44760603 | A | 0.215 | 0.0197 | 1.00 | 0.287 | 7.86E-28 |
| rs35796750 | 21:47422412 | T | -0.1246 | 0.0208 | 1.00 | 0.455 | 2.05E-09 |
| rs12628032 | 22:19967980 | T | 0.2277 | 0.0224 | 0.30 | 0.309 | 2.65E-24 |
| rs9608690 | 22:28921347 | A | -0.3711 | 0.06 | 1.00 | 0.068 | 6.13E-10 |
| rs4823006 | 22:29451671 | A | 0.1396 | 0.0174 | 0.95 | 0.445 | 1.17E-15 |
| rs5753103 | 22:30768777 | A | 0.1377 | 0.0206 | 1.00 | 0.444 | 2.62E-11 |
| rs9609429 | 22:32517431 | T | 0.1203 | 0.0195 | 0.99 | 0.284 | 6.32E-10 |
| rs470113 | 22:40729614 | A | -0.2403 | 0.0265 | 0.82 | 0.182 | 1.43E-19 |
| rs73161324 | 22:42038786 | T | 0.3112 | 0.0478 | 0.50 | 0.056 | 7.55E-11 |

Abbreviations: Chr, chromosome; INFO, imputation quality score; and MAF, minor allele frequency.

**Table S2.** Descriptive characteristics of participants in the UK Biobank study by hypertension.

| Characteristic | Category | | |
| --- | --- | --- | --- |
|  | Origin^*^  (n = 502,507) | Baseline hypertension^**^ (n = 437,185) | Missing covariate^***^  (n = 25,487) |
| Age (years, mean ± SD) | 56.5 ± 8.1 | 56.0 ± 8.1 | 56.9 ± 8.1 |
| Sex, male (n, %) | 229,123 (45.6) | 194,302 (44.4) | 70,930 (50.9) |
| Race, White (n, %) | 472,128 (94.1) | 411,698 (94.2) | - |
| TDI (mean ± SD) | -1.3 ± 3.1 | -1.4 ± 3.1 | -1.0 ± 3.3 |
| BMI (kg/m^2^, mean ± SD) | 27.4 ± 4.8 | 27.2 ± 4.7 | 27.4 ± 4.6 |
| BMI (kg/m^2^, n, %) |  |  |  |
| Normal (<25 kg/m^2^) | 165,036 (32.9) | 151,279 (34.6) | 7,929 (31.1) |
| Overweight (25 to 29.9 kg/m^2^) | 212,118 (42.2) | 185,474 (42.4) | 10,619 (41.7) |
| Obesity (≥30 kg/m^2^) | 122,248 (24.3) | 98,315 (22.5) | 6,472 (25.4) |
| Missing value | 3,105 (0.6) | 2,117 (0.5) | 467 (1.8) |
| Physical activity (MET, Min/week, mean ± SD) | 2650.2 ± 2713.1 | 2666.9 ± 2710.7 | 2633.8 ± 2743.3 |
| Smoke status (n, %) |  |  |  |
| Never | 273,522 (54.4) | 243,723 (55.7) | 17,320 (67.9) |
| Previous | 173,058 (34.4) | 146,410 (33.5) | 4,860 (19.1) |
| Current | 52,979 (10.4) | 45,541 (10.4) | 3,089 (12.1) |
| Missing value | 2,948 (0.6) | 1,511 (0.4) | 218 (0.9) |
| Alcohol drinker status (n, %) |  |  |  |
| Never | 22,385 (4.5) | 18,946 (4.3) | 6,202 (24.3) |
| Previous | 18,104 (3.6) | 14,567 (3.3) | 1,316 (5.2) |
| Current | 460,365 (91.6) | 403,197 (92.2) | 17,801 (69.8) |
| Missing value | 1,653 (0.3) | 475 (0.2) | 168 (0.7) |
| Diabetes baseline (n, %) | 26,400 (5.3) | 17,741 (4.1) | 2,454 (9.6) |
| Maternal smoking around birth (n, %) | 126625 (25.6) | 116,286 (29.1) | 2,117 (8.8) |
| Breasted as a baby (n, %) | 277598 (55.34) | 256,455 (72.0) | 19,447 (88.3) |

Data are presented as the mean ± standard deviation (SD), numbers and percentages.

Abbreviations: TDI, Townsend Deprivation index; BMI, body mass index; and MET, Metabolic Equivalent Task.

* The total of 502,507 people

**Participants without baseline hypertension

***Participants who missing the data of covariates

**Table S3.** The association of breastfeeding and maternal smoking on hypertension.

| Maternal smoking | Breastfeeding | Case/control | HR (95% CI) | *P* |
| --- | --- | --- | --- | --- |
| Yes | No | 4,082/26,995 | ref | - |
|  | Yes | 9,607/48,412 | 0.97 (0.93, 1.01) | 0.124 |
| No | No | 6,697/50,013 | ref | - |
|  | Yes | 26,057/146,562 | 0.97 (0.94, 1.00) | 0.084 |

Abbreviations: HR, hazard ratio; and CI, confidence interval.

Adjusted for age (continuous), sex (male, female), race (White/Mixed/Asian or Asian British/Black or Black British), UK Biobank assessment centre, Townsend Deprivation index (continuous), alcohol consumption (never, previous, current, missing), smoking status (never, previous, current, missing), body mass index (<25 kg/m^2^, 25 to 29.9 kg/m^2^, ≥30 kg/m^2^, missing), physical activity (continuous), and diabetes at baseline (yes/no).

**Table S4.** Subgroup analysis for the association of hypertension and maternal smoking or breastfeeding by specific characteristics.

| Characteristic | Maternal smoking (n = 399,531) | | | Breastfeeding (n = 356,079) | | |
| --- | --- | --- | --- | --- | --- | --- |
|  | Case/control | HR (95% CI) | *P*^*^ | Case/control | HR (95% CI) | *P*^*^ |
| Age |  |  | <0.001 |  |  | 0.013 |
| <60 | 600/259,685 | 1.17 (1.13, 1.20) |  | 650/279,659 | 1.10 (1.06, 1.13) |  |
| >=60 | 658/200,186 | 1.03 (1.01, 1.06) |  | 716/214,062 | 1.02 (0.98, 1.05) |  |
| Sex |  |  | 0.061 |  |  | 0.683 |
| Female | 412/249,937 | 1.09 (1.06, 1.12) |  | 446/268,804 | 0.96 (0.93, 0.99) |  |
| Male | 846/209,934 | 1.12 (1.09, 1.15) |  | 920/224,917 | 0.97 (0.93, 1.00) |  |
| BMI |  |  | 0.470 |  |  | 0.614 |
| Normal (<25 kg/m^2^) | 260/150,879 | 1.11 (1.06, 1.16) |  | 286/162,135 | 0.94 (0.89, 0.99) |  |
| Overweight (25 to 29.9 kg/m^2^) | 452/194,407 | 1.10 (1.07, 1.13) |  | 488/208,776 | 0.98 (0.95, 1.02) |  |
| Obesity (≥30 kg/m^2^) | 526/112,085 | 1.10 (1.06, 1.13) |  | 572/120,195 | 0.96 (0.92, 1.00) |  |
| Smoke status |  |  | 0.238 |  |  | 0.818 |
| Never | 479/250,769 | 1.10 (1.07, 1.13) |  | 514/269,633 | 0.98 (0.94, 1.01) |  |
| Previous | 494/159,542 | 1.10 (1.07, 1.14) |  | 529/170,350 | 0.94 (0.91, 0.98) |  |
| Current | 275/47,658 | 1.15 (1.09, 1.22) |  | 312/51,726 | 0.96 (0.89, 1.03) |  |

Abbreviations: BMI, body mass index; HR, hazard ratio; and CI, confidence interval.

Adjusted for age (continuous), sex (male, female), race (White/Mixed/Asian or Asian British/Black or Black British), UK Biobank assessment centre, Townsend Deprivation index (continuous), alcohol consumption (never, previous, current, missing), smoking status (never, previous, current, missing), body mass index (<25 kg/m^2^, 25 to 29.9 kg/m^2^, ≥30 kg/m^2^, missing), physical activity (continuous), and diabetes at baseline (yes/no). The stratified factor in each stratum was excluded.

* *P* value for interaction.

**Table S5.** The association of maternal smoking and sex with hypertension by sex (n = 399,531).

| Maternal smoking | Sex | Case/control | HR (95% CI) | *P* |
| --- | --- | --- | --- | --- |
| No | women | 20,797/137,830 | ref | - |
|  | men | 22,213/102,405 | 1.26 (1.23, 1.29) | <2E-16 |
| Yes | women | 8,726/54,167 | 1.08 (1.05, 1.12) | 5.57E-08 |
|  | men | 10,404/42,989 | 1.42 (1.38, 1.46) | <2E-16 |

Adjusted for age (continuous), race (White/Mixed/Asian or Asian British/Black or Black British), UK Biobank assessment centre, Townsend Deprivation index (continuous), alcohol consumption (never, previous, current, missing), smoking status (never, previous, current, missing), body mass index (<25 kg/m^2^, 25 to 29.9 kg/m^2^, ≥30 kg/m^2^, missing), physical activity (continuous), and diabetes at baseline (yes/no).

**Table S6.** The association of maternal smoking and own smoking history with hypertension (n = 318,425).

| Maternal smoking | Smoking history | Case/control | HR (95% CI) | *P** | *P*** |
| --- | --- | --- | --- | --- | --- |
| No | Never | 21,581/140,610 | ref | - | 0.070 |
|  | Former | 17,039/76,386 | 1.12 (1.09, 1.15) | <2E-16 |  |
|  | Current | 4,180/22,429 | 1.22 (1.18, 1.27) | <2E-16 |  |
| Yes | Never | 8,928/54,065 | 1.09 (1.06, 1.13) | 3.97E-10 |  |
|  | Former | 7,665/31,184 | 1.24 (1.20, 1.27) | <2E-16 |  |
|  | Current | 2,448/11,666 | 1.43 (1.37, 1.51) | <2E-16 |  |

Adjusted for age (continuous), sex (male, female), race (White/Mixed/Asian or Asian British/Black or Black British), UK Biobank assessment centre, Townsend Deprivation index (continuous), alcohol consumption (never, previous, current, missing), body mass index (<25 kg/m^2^, 25 to 29.9 kg/m^2^, ≥30 kg/m^2^, missing), physical activity (continuous), and diabetes at baseline (yes/no).

* Stratification analysis

** The analysis of interaction between smoking during pregnancy and self-smoking

| Maternal smoking | Smoking history | Women (n= 220,785) | | | Men (n= 177,396) | | | |
| --- | --- | --- | --- | --- | --- | --- | --- | --- |
|  |  | Case/control | HR (95% CI) | *P** | Case/control | HR (95% CI) | *P** | *P*** |
| No | Never | 12,307/86,388 | ref | - | 9,274/54,222 | ref | - | 0.014 |
|  | Former | 6,784/40,358 | 1.07 (1.03, 1.10) | 3.88E-04 | 10,255/36,028 | 1.18 (1.14, 1.22) | <2E-16 |  |
|  | Current | 1,598/10,646 | 1.21 (1.14, 1.29) | 1.83E-09 | 2,582/11,783 | 1.25 (1.19, 1.31) | <2E-16 |  |
| Yes | Never | 4,563/31,561 | 1.07 (1.02, 1.11) | 1.87E-03 | 4,365/22,504 | 1.13 (1.08, 1.17) | 3.10E-09 |  |
|  | Former | 3,126/16,768 | 1.18 (1.13, 1.24) | 6.14E-13 | 4,539/14,416 | 1.30 (1.24, 1.35) | <2E-16 |  |
|  | Current | 992/5,694 | 1.42 (1.31, 1.54) | <2E-16 | 1,456/5,972 | 1.46 (1.37, 1.56) | <2E-16 |  |

**Table S7.** The association of maternal smoking and own smoking history with hypertension by sex (n = 398,181).

Adjusted for age (continuous), race (White/Mixed/Asian or Asian British/Black or Black British), UK Biobank assessment centre, Townsend Deprivation index (continuous), alcohol consumption (never, previous, current, missing), body mass index (<25 kg/m^2^, 25 to 29.9 kg/m^2^, ≥30 kg/m^2^, missing), physical activity (continuous), and diabetes at baseline (yes/no).

* Stratification analysis

** The analysis of interaction between sex and own smoking history

**Table S8.** Adjusted hazard ratios and 95% confidence intervals for hypertension polygenic risk score with the risk of hypertension (n = 400,124).

|  | Hypertension PRS (tertiles) | | | | |
| --- | --- | --- | --- | --- | --- |
|  | Low | Intermediate | High | Per unit of PRS | *P* |
| Case/control | 15,801/110,806 | 20,462/114,975 | 25,438/112,642 |  |  |
| Model 1 | Ref | 1.25 (1.22, 1.27) | 1.57 (1.54, 1.60) | 1.07 (1.06, 1.07) | <2E-16 |
| Model 2 | Ref | 1.25 (1.23, 1.28) | 1.1 (1.57, 1.64) | 1.07 (1.07, 1.07) | <2E-16 |

PRS, polygenic risk score.

Model 1, age (continuous), sex (male, female);

Model 2, Adjusted for age (continuous), sex (male, female), UK Biobank assessment centre, Townsend Deprivation index (continuous), alcohol consumption (never, previous, current, missing), smoking status (never, previous, current, missing), body mass index (<25 kg/m^2^, 25 to 29.9 kg/m^2^, ≥30 kg/m^2^, missing), physical activity (continuous), diabetes at baseline (yes/no), genotyping batch, and the first 4 genetic principal components.

**Table S9.** The joint association of maternal smoking and breastfeeding on incident hypertension in participants with different genetic risk after excluding participants with cardiovascular disease at baseline (n = 283,057).

| Hypertension PRS (tertiles) | Category of exposure | HR (95% CI) | *P* | *P* for interaction |
| --- | --- | --- | --- | --- |
|  | Maternal smoking |  |  |  |
| Low genetic risk | No | Ref | Ref | 0.355 |
|  | Yes | 1.13 (1.08, 1.18) | 6.10E-07 |  |
| Intermediate genetic risk | No | 1.30 (1.25, 1.34) | <0.001 |  |
|  | Yes | 1.39 (1.33, 1.45) | <0.001 |  |
| High genetic risk | No | 1.67 (1.61, 1.72) | <0.001 |  |
|  | Yes | 1.82 (1.74, 1.89) | <0.001 |  |
|  | Breastfeeding |  |  |  |
| Low genetic risk | Yes | Ref | Ref | 0.524 |
|  | No | 1.03 (0.98, 1.09) | 0.234 |  |
| Intermediate genetic risk | Yes | 1.26 (1.22, 1.30) | <0.001 |  |
|  | No | 1.37 (1.31, 1.44) | <0.001 |  |
| High genetic risk | Yes | 1.65 (1.60, 1.71) | <0.001 |  |
|  | No | 1.69 (1.61, 1.76) | <0.001 |  |

Abbreviation: HR: hazard rations; CI, confidence interval; PRS, polygenic risk score.

^*^ Adjusted for age (continuous), sex (male, female), UK Biobank assessment center, Townsend Deprivation index (continuous), alcohol consumption (never, previous, current, missing), smoking status (never, previous, current, missing), body mass index (<25 kg/m^2^, 25 to 29.9 kg/m^2^, ≥30 kg/m^2^, missing), physical activity (continuous), and diabetes at baseline (yes/no), genotyping batch, and the first 4 genetic principal components.

**Table S10.** The joint association of maternal smoking and breastfeeding with incident hypertension in participants with different genetic risk after excluding participants with follow-up time of less than 2 years in the UK Biobank (n = 278,873).

| Hypertension PRS (tertiles) | Category of exposure | HR (95% CI) | *P* | *P* for interaction |
| --- | --- | --- | --- | --- |
|  | Maternal smoking |  |  |  |
| Low genetic risk | No | Ref | Ref | 0.578 |
|  | Yes | 1.11 (1.06, 1.17) | 4.30E-05 |  |
| Intermediate genetic risk | No | 1.27 (1.22, 1.32) | <0.001 |  |
|  | Yes | 1.40 (1.33, 1.47) | <0.001 |  |
| High genetic risk | No | 1.64 (1.58, 1.71) | <0.001 |  |
|  | Yes | 1.80 (1.72, 1.88) | <0.001 |  |
|  | Breastfeeding |  |  |  |
| Low genetic risk | Yes | Ref | Ref | 0.993 |
|  | No | 1.02 (0.97, 1.08) | 0.423 |  |
| Intermediate genetic risk | Yes | 1.25 (1.20, 1.30) | <0.001 |  |
|  | No | 1.35 (1.28, 1.42) | <0.001 |  |
| High genetic risk | Yes | 1.63 (1.57, 1.69) | <0.001 |  |
|  | No | 1.68 (1.61, 1.77) | <0.001 |  |

Abbreviation: HR: hazard rations; CI, confidence interval; PRS, polygenic risk score.

Adjusted for age (continuous), sex (male, female), UK Biobank assessment center, Townsend Deprivation index (continuous), alcohol consumption (never, previous, current, missing), smoking status (never, previous, current, missing), body mass index (<25 kg/m^2^, 25 to 29.9 kg/m^2^, ≥30 kg/m^2^, missing), physical activity (continuous), and diabetes at baseline (yes/no), genotyping batch, and the first 4 genetic principal components.

**Table S11.** The joint association of maternal smoking and breastfeeding with incident hypertension in participants with different genetic risk among never smokers (n = 162,439).

| Hypertension PRS (tertiles) | Category of exposure | HR (95% CI) | *P* | *P* for interaction |
| --- | --- | --- | --- | --- |
|  | Maternal smoking |  |  |  |
| Low genetic risk | No | Ref | Ref | 0.756 |
|  | Yes | 1.09 (1.02, 1.17) | 0.011 |  |
| Intermediate genetic risk | No | 1.29 (1.23, 1.36) | <0.001 |  |
|  | Yes | 1.40 (1.32, 1.49) | <0.001 |  |
| High genetic risk | No | 1.67 (1.60, 1.75) | <0.001 |  |
|  | Yes | 1.80 (1.70, 1.91) | <0.001 |  |
|  | Breastfeeding |  |  |  |
| Low genetic risk | Yes | Ref | Ref | 0.559 |
|  | No | 0.99 (0.92, 1.07) | 0.804 |  |
| Intermediate genetic risk | Yes | 1.28 (1.22, 1.34) | <0.001 |  |
|  | No | 1.32 (1.24, 1.41) | <0.001 |  |
| High genetic risk | Yes | 1.65 (1.58, 1.73) | <0.001 |  |
|  | No | 1.69 (1.59, 1.80) | <0.001 |  |

Abbreviation: HR: hazard rations; CI, confidence interval; PRS, polygenic risk score.

^*^ Adjusted for age (continuous), sex (male, female), UK Biobank assessment center, Townsend Deprivation index (continuous), alcohol consumption (never, previous, current, missing), smoking status (never, previous, current, missing), body mass index (<25 kg/m^2^, 25 to 29.9 kg/m^2^, ≥30 kg/m^2^, missing), physical activity (continuous), and diabetes at baseline (yes/no), genotyping batch, and the first 4 genetic principal components.


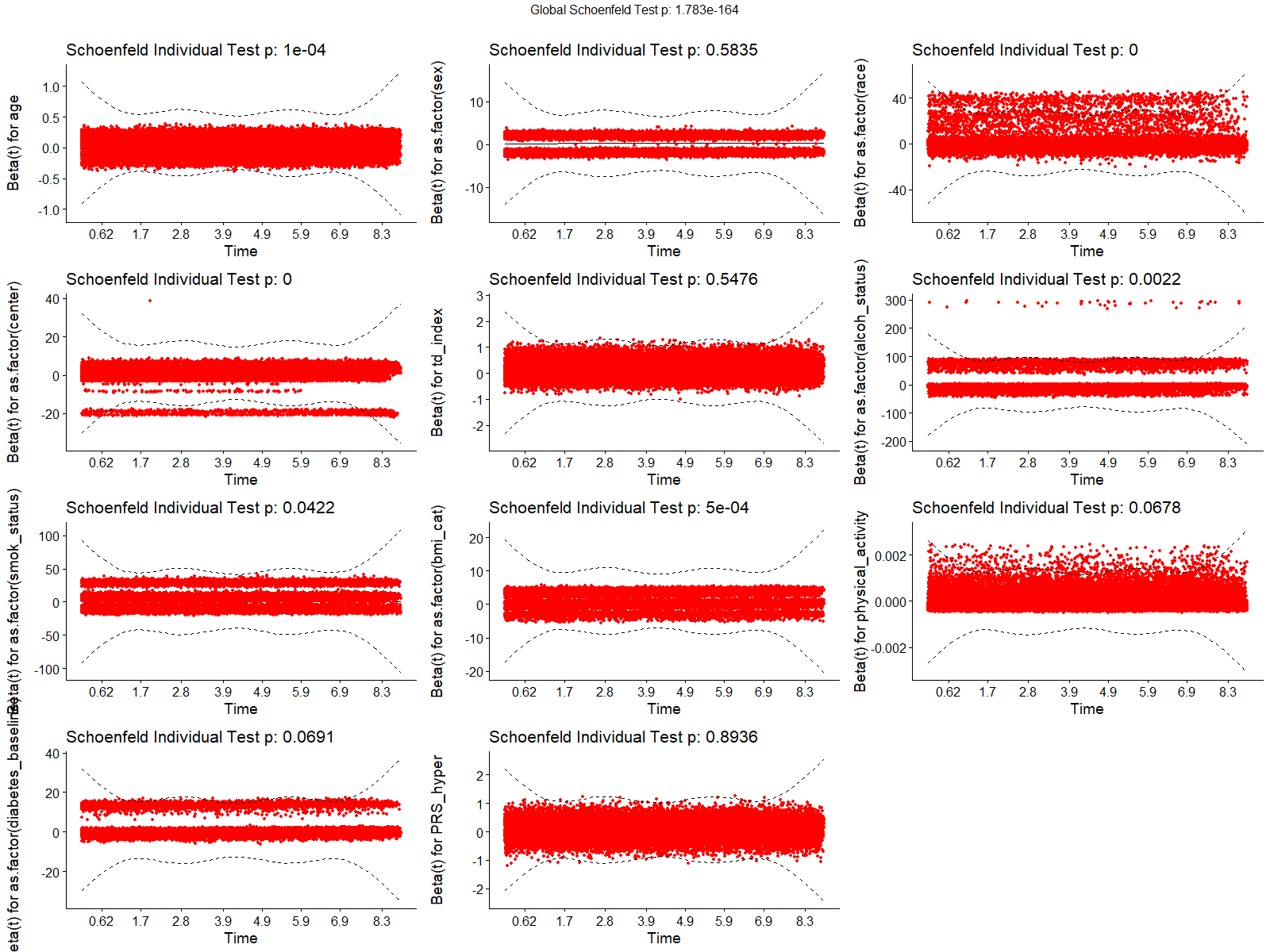
**Figure S1**. The proportional hazards assumption using Schoenfeld residuals.
